# Heavy mechanical force decelerates orthodontic tooth movement via Piezo1-induced mitochondrial calcium down-regulation

**DOI:** 10.1016/j.gendis.2024.101434

**Published:** 2024-09-15

**Authors:** Ye Zhu, Xuehuan Meng, Qiming Zhai, Liangjing Xin, Hao Tan, Xinyi He, Xiang Li, Guoyin Yang, Jinlin Song, Leilei Zheng

**Affiliations:** College of Stomatology, Chongqing Medical University, Chongqing Key Laboratory of Oral Diseases, Chongqing Municipal Key Laboratory of Oral Biomedical Engineering of Higher Education, Chongqing 401147, China

**Keywords:** Biomechanics, Bone remodeling/regeneration, Mechanotransduction, Orthodontic tooth movement, Periodontal ligament

## Abstract

Orthodontic tooth movement (OTM) depends on periodontal ligament cells (PDLCs), which sense biomechanical stimuli and initiate alveolar bone remodeling. Light (optimal) forces accelerate OTM, whereas heavy forces decelerate it. However, the mechanisms by which PDLCs sense biomechanical stimuli and affect osteoclastic activities under different mechanical forces (MFs) remain unclear. This study demonstrates that mechanosensitive ion channel Piezo1-mediated Ca^2+^ signal conversion is crucial for sensing and delivering biomechanical signals in PDLCs under heavy-force conditions. Heavy MF up-regulated Piezo1 in PDLCs, reducing mitochondrial Ca^2+^ influx by inhibiting ITPR3 expression in mitochondria-associated membranes. Decreased mitochondrial calcium uptake led to reduced cytoplasmic release of mitochondrial DNA and inhibited the activation of the cGAS‒STING signaling cascade, subsequently inhibiting monocyte-to-osteoclast differentiation. Inhibition of Piezo1 or up-regulation of STING expression under heavy MF conditions significantly increased osteoclast activity and accelerated OTM. These findings suggest that heavy MF-induced Piezo1 expression in PDLCs is closely related to the control of osteoclast activity during OTM and plays an essential role in alveolar bone remodeling. This mechanism may be a potential therapeutic target for accelerating OTM.

## Introduction

Orthodontic tooth movement (OTM) is induced by orthodontic mechanical force (MF) stimuli and is dependent on periodontal tissue remodeling.^1^^,^^2^ Periodontal ligament cells (PDLCs) connect alveolar bone and teeth, directly sensing MFs and regulating osteoclast activity, which plays a crucial role in alveolar bone remodeling and OTM.^3^ However, heavy MFs can cause damage to periodontal tissues, leading to the deregulation of downstream osteoclast activity during OTM.^4^^,^^5^ The mechanisms by which PDLCs sense biomechanical stimuli and influence osteoclast activity under light or heavy MF conditions remain insufficiently understood.

Piezo1, a mechanosensitive ion channel, is vital for mediating Ca^2+^ signal conversion in response to MF stimuli.^6^ Piezo1 is more sensitive to heavy MF stimuli than to light MF stimuli.^7^ Numerous studies have demonstrated that Piezo1 regulates inflammation and osteoclast activity during OTM in PDLCs.^8^^,^^9^ However, the mechanism by which Piezo1 regulates Ca^2+^ signaling to inhibit downstream osteoclast activity under heavy MF stress remains unclear.

Extracellular Ca^2+^ is taken up by Piezo1, enters the cell as a second messenger, and is involved in intracellular Ca^2+^ homeostasis.^10^^,^^11^ Mitochondria play a pivotal role in cell fate, partially because of their involvement in the dynamic regulation of cellular Ca^2+^.^12^ Excessive mitochondrial Ca^2+^ ([Ca^2+^]_m_) uptake promotes inflammation, whereas reduced [Ca^2+^]_m_ content may impair cellular bioenergetics and contribute to cellular dysfunction and apoptosis.^13^ The Ca^2+^ channel between the endoplasmic reticulum (ER) and mitochondria, known as the mitochondria-associated ER membrane (MAM), plays a crucial role in regulating [Ca^2+^]_m_ homeostasis.^14^ Studies suggest a close correlation between [Ca^2+^]_m_ homeostasis and biomechanical stimuli, but how light or heavy MF-activated Piezo1 affects [Ca^2+^]_m_ homeostasis in OTM and alveolar bone remodeling remains unknown.

This study demonstrated that, unlike light MF, heavy MF activated Piezo1 and decreased [Ca^2+^]_m_ levels by down-regulating ITPR3 (inositol 1,4,5-trisphosphate receptor type 3) expression in PDLCs. The low level of [Ca^2+^]_m_ under heavy MF conditions led to a decrease in cytoplasmic release of mitochondrial DNA (mtDNA) and a corresponding decrease in cGAS–STING signaling pathway activity, thereby down-regulating alveolar bone remodeling. Our study suggests an effective strategy for OTM and that modulating inflammation through Piezo1 is a promising potential avenue for enhancing the speed of OTM.

## Materials and methods

### Orthodontic loading *in vivo*

All animal experiments were approved by the Institutional Animal Care and Use Committee of Chongqing Medical University (No. 202310171728000204352). An OTM animal model was established as previously described.^15^^,^^16^ A nickel‒titanium coil spring with 25 g (light MF) or 100 g (heavy MF) was fixed between the maxillary left first molar and the incisors to induce orthodontic tooth movement.^16^ The appliances were immediately activated upon insertion and their fit was assessed daily. No reactivation was conducted throughout the experiment.

### Local injection intervention in animal models

Six-week-old male Sprague–Dawley rats were obtained from the Experimental Animal Experiment Center of Chongqing Medical University. The animals were raised in a specific pathogen-free environment and then randomly allocated into six groups, three per group, as follows: (i) Light group: optimal orthodontic MF; (ii) Heavy group: heavy orthodontic MF; (iii) Yoda1-Light group: optimal orthodontic MF + Yoda1 (Piezo1 agonist; dosage, 20 μL; concentration, 5 μM; MedChemExpress, United States); (iv) GsMTx4-Heavy group: heavy orthodontic MF + GsMTx4 (Piezo1 inhibitor; dosage, 20 μL; concentration, 3 μM; MedChemExpress); (v) C-176-Light group: optimal orthodontic MF + C176 (STING inhibitor; dosage, 20 μL; concentration, 0.5 μM; MedChemExpress); and (vi) ADU-S100-Heavy group: heavy orthodontic MF + ADU-S100 (STING agonist; dosage, 20 μL; concentration, 5 μM; MedChemExpress). The animals were administered a local injection of GsMTx4, C-176, Yoda1, or ADU-S100 every other day. Each group was euthanized on days 1, 3, and 7 following tooth movement. The alveolar bone blocks included the left first molar and were harvested for subsequent analysis.

### Cell culture

Periodontal ligament (PDL) tissues were extracted from the teeth of patients at the Stomatological Hospital of Chongqing Medical University who required extraction during orthodontic treatment. PDLCs were extracted as previously described.^17^^,^^18^

### Orthodontic MF loading *in vitro*

PDLCs were subjected to static compressive MF via the static compression technique. The concentrations of the optimal light MF (2 g/cm^2^) and heavy MF (8 g/cm^2^) were determined according to previous studies.^17^ Briefly, a glass cylinder was placed over a confluent cell layer. The magnitudes of compression force were set as 2 g/cm^2^ (light force) and 8 g/cm^2^ (heavy force) as a compression force of 2.2 g/cm^2^ previously demonstrated to be the physiological magnitude at which cells trigger biological responses.^19^^,^^20^ Our results revealed that when the MF reached 8 g/cm^2^, the cytoskeleton of the PDLCs began to shrink, the proliferation ability significantly decreased, and cell apoptosis occurred (Fig. S1).

Cells cultured in the light force served as the control group. According to a previous study, 6 h loading is sufficient for PDLCs to sense mechanical stimuli and activate downstream cellular responses and biological events; thus, a mechanical loading time of 6 h was used.^21^

### *In vitro* orthodontic MF loading intervention

The groups in this study were categorized as follows: (i) Light group: subjected to light MF for PDLCs; (ii) Heavy group: subjected to heavy MF for PDLCs; (iii) Yoda1-Light group: subjected to light MF for PDLCs with Yoda1 (5 μM) treatment; (iv) GsMTx4-Heavy group: subjected to heavy MF for PDLCs with GsMTx4 (4 μM) treatment; (v) siITPR3-Light group: subjected to light MF for PDLCs after ITPR3 small-interfering RNA treatment; (vi) siITPR3-Heavy group: subjected to heavy MF for PDLCs after ITPR3 small-interfering RNA treatment; (vii) C-176-Light group: subjected to light MF for PDLCs with C-176 (20 μM) treatment; (viii) ADU-S100-Heavy group: subjected to heavy MF for PDLCs with ADU-S100 (10 μM) treatment.

## Results

### Heavy MF activation of Piezo1 decelerates OTM

**Figure 1:**
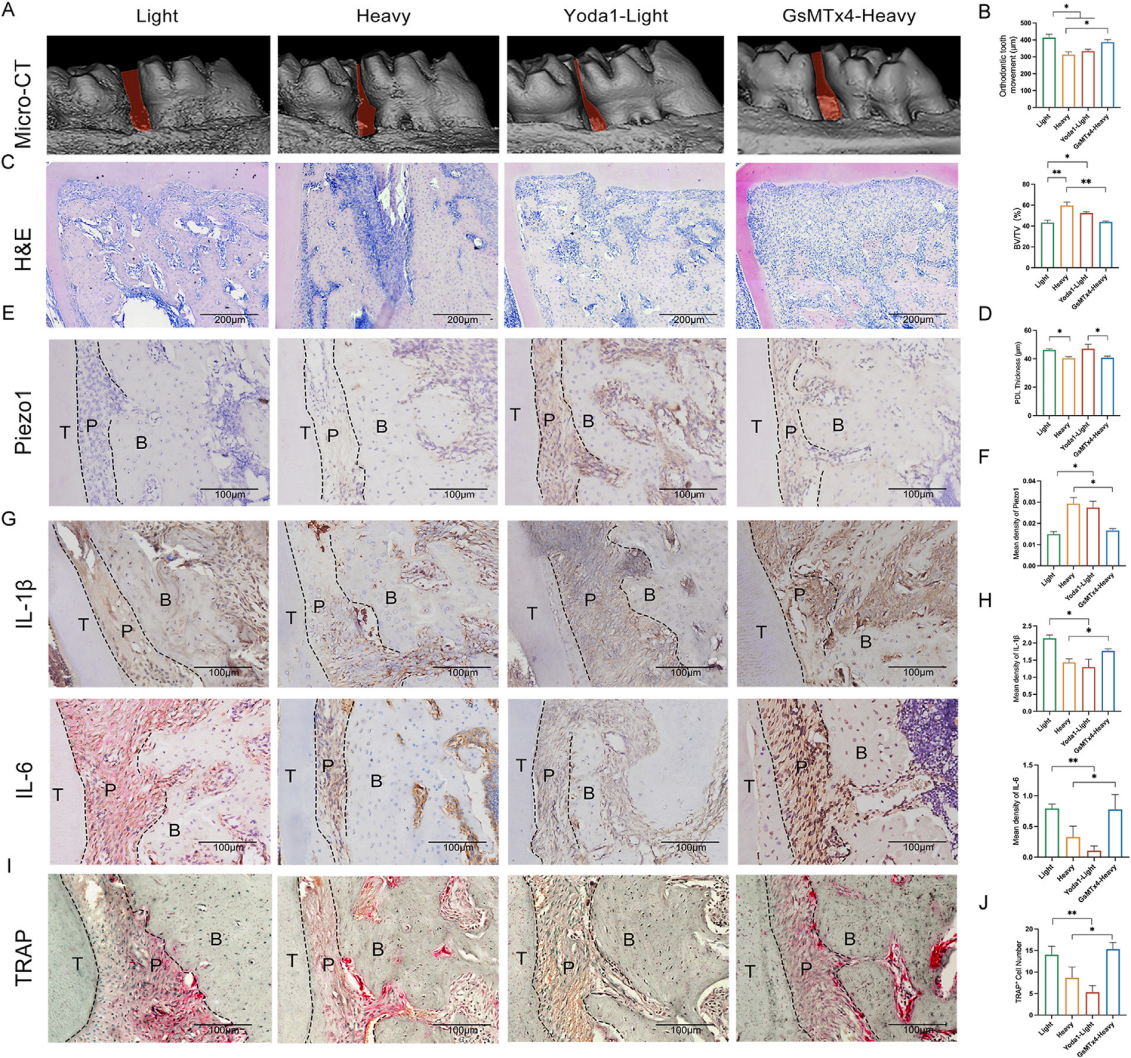
Heavy mechanical force (MF)-activated Piezo1 decelerates orthodontic tooth movement (OTM) *in vivo*. **(A, B)** Micro-CT analysis of OTM at 7 days after intervention with Piezo1 agonist (Yoda1) or inhibitor (GsMTX4). **(C, D)** Hematoxylin‒eosin staining of orthodontic teeth from rats treated with Piezo1 agonist (Yoda1) or inhibitor (GsMTX4). Scale bar, 200 μm. **(E, F)** Immunohistochemical staining of Piezo1 after treatment with Piezo1 agonist (Yoda1) or inhibitor (GsMTX4) for 1 day. Scale bar, 100 μm. **(G, H)** Immunohistochemical staining of IL-1β and IL-6 after intervention with Piezo1 agonist (Yoda1) or inhibitor (GsMTX4) for 7 days. Scale bar, 100 μm. **(I, J)** Tartrate-resistant acid phosphatase staining after treatment with Piezo1 agonist (Yoda1) and inhibitor (GsMTX4). Scale bar, 100 μm.

The differences in OTM under light or heavy MF conditions were assessed *in vivo*. Micro-CT scans and analyses indicated that after 7 days, the OTM distance of the Heavy group decreased (Fig. 1A; Fig. S2, 3). The corresponding ratio of the trabecular bone volume over the total cancellous tissue volume was greater in the heavy MF groups than in the light groups. To investigate the role of Pizeo1 in the regulation of OTM by MF, we treated the mechanosensitive ion channel Pizeo1 with both a selective inhibitor (GsMTx4) and an agonist (Yoda1) at an appropriate dosage. Micro-CT data demonstrated that GsMTx4 promoted OTM and bone remodeling under heavy MF conditions, whereas Yoda1 inhibited OTM and bone remodeling under light MF conditions (Fig. 1B). Hematoxylin & eosin staining (Fig. 1C; Fig. S4) revealed that both light and heavy MFs significantly reduced the thickness of the PDL on the pressure side at 1 day (Fig. 1D). These results suggest that the OTM model was successfully established and that MF regulates OTM and alveolar bone remodeling through Piezo1.

Immunohistochemical staining for Piezo1 (Fig. 1E, F; Fig. S5) revealed a notable increase in Piezo1 expression in the PDL after heavy MF exposure (Fig. 1F). Immunohistochemical staining of interleukin (IL)-1β and IL-6 (Fig. 1G; Fig. S6, 7) revealed that GsMTx4 promoted IL-1β and IL-6 expression in PDLs under heavy MF conditions, whereas Yoda1 inhibited IL-1β and IL-6 expression in PDLs under light MF conditions (Fig. 1H). Tartrate-resistant acid phosphatase (TRAP) staining (Fig. 1I, J; Fig. S8) revealed a similar trend of positive cell numbers, which correlated with IL-1β and IL-6 expression. These results indicate that heavy MF slows OTM and reduces pressure-side osteoclast remodeling levels by activating Piezo1.

### Heavy MF-activated Piezo1 inhibits ITPR3 expression *in vitro*

**Figure 2:**
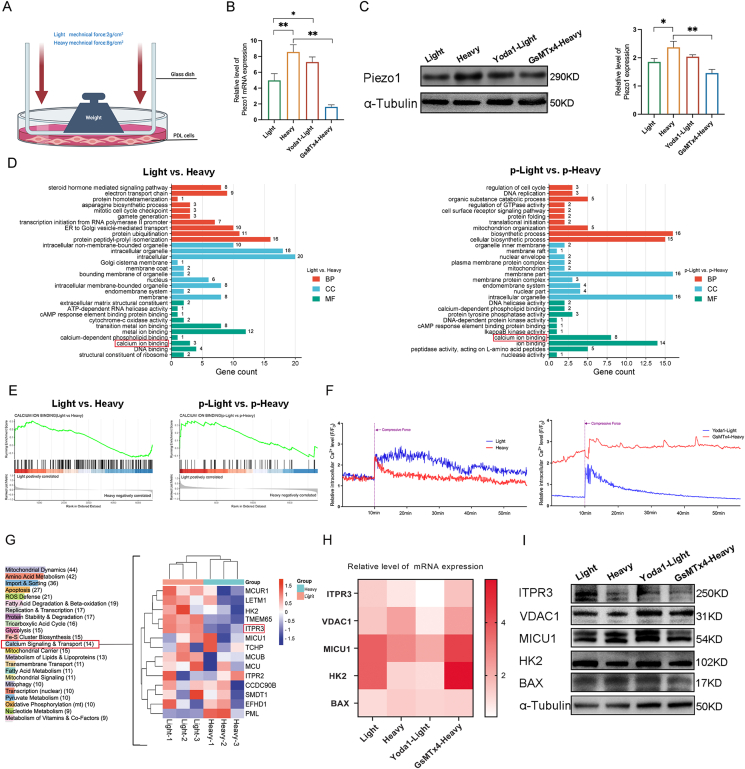
Heavy mechanical force (MF) conditions activate Piezo1-inhibited ITPR3 in periodontal ligament stem cells. **(A)** Model schematic diagram of the MF applied to periodontal ligament stem cells *in vitro*. **(B, C)** Quantitative reverse-transcription PCR (B) and Western blot analysis (C) of Piezo1 under light or heavy MF conditions. **(D)** Gene Ontology analysis of the proteome (Light *vs*. Heavy) and phosphorylation-modified proteome (p-Light *vs*. p-Heavy) under light and heavy MF stress. BP, biological process; CC, cellular component; MF, molecular function. **(E)** GSEA enrichment of the proteome- and phosphorylation-modified proteome under light or heavy MF stress. **(F)** Ca^2+^ oscillation analysis under light or heavy MF stress. **(G)** mitoXplorer 2.0 analysis of the proteome. **(H, I)** Quantitative reverse-transcription PCR and Western blot analysis of mitochondria-associated membrane-related calcium signaling protein expression. ITPR3, inositol 1,4,5-trisphosphate receptor type 3.

To illustrate the expression of Piezo1 in response to light or heavy MF exposure *in vitro*, we utilized an MF application model to expose PDLCs to either light or heavy MF (Fig. 2A). Quantitative reverse-transcription PCR (Fig. 2B), western blotting (Fig. 2C), and immunofluorescence (Fig. S9) assays collectively confirmed that Piezo1 was significantly increased in PDLCs under heavy MF conditions compared with those under light MF conditions. This finding aligns with the immunohistochemical results for Piezo1 expression *in vivo*.

We then subjected PDLCs to light or heavy MF analysis via proteomics and phosphorylation proteomics. Significant differences in the expression of calcium ion binding and calcium signaling pathway-related proteins were observed between the Light and Heavy groups, as revealed through Gene Ontology (GO) term analysis (Fig. 2D). Proteome gene set enrichment analysis (GSEA) also revealed that calcium ion binding in PDLCs was significantly up-regulated under light MF conditions but down-regulated under heavy MF conditions (Fig. 2E).

To further assess the intracellular calcium ([Ca^2+^]_i_) levels in PDLCs under light or heavy MF conditions, Fluo8-AM was used to label [Ca^2+^]_i_ (Fig. 2F). Notably, [Ca^2+^]_i_ levels were significantly lower under heavy MF conditions than under light MF conditions. Furthermore, the activation of Piezo1 with Yoda1 treatment significantly decreased [Ca^2+^]_i_ levels under light MF conditions, whereas the inhibition of Piezo1 with GsMTx4 treatment substantially elevated [Ca^2+^]_i_ levels under heavy MF conditions (Fig. 2F). Next, we employed an analysis platform, MitoXplorer 2.0,^22^ to investigate Ca^2+^ homeostasis in PDLCs under light or heavy MF conditions (Fig. 2G). Calcium signaling and transport enrichment revealed that the expression of ITPR3, a calcium transporter located in MAMs, was specifically up-regulated under light MF conditions. This observation was validated by quantitative reverse-transcription PCR and Western blot analysis (Fig. 2H, I; Fig. S10A, B). To explore the potential regulation of ITPR3 by Piezo1 through extracellular Ca^2+^ influx, we exposed PDLCs to different Ca^2+^ concentrations. Western blot analysis revealed that depletion of [Ca^2+^]_i_ had minimal effects on ITPR3 expression in both the Light and Heavy groups (Fig. S10C). However, deprivation of Ca^2+^ from the extracellular medium notably decreased the expression of ITPR3 (Fig. S10D). These findings suggest a key role of extracellular Ca^2+^ uptake via Piezo1 in the response to MF stress.

### Heavy MF-activated Piezo1 down-regulates [Ca^2+^]_m_ levels by inhibiting ITPR3 in MAMs

**Figure 3:**
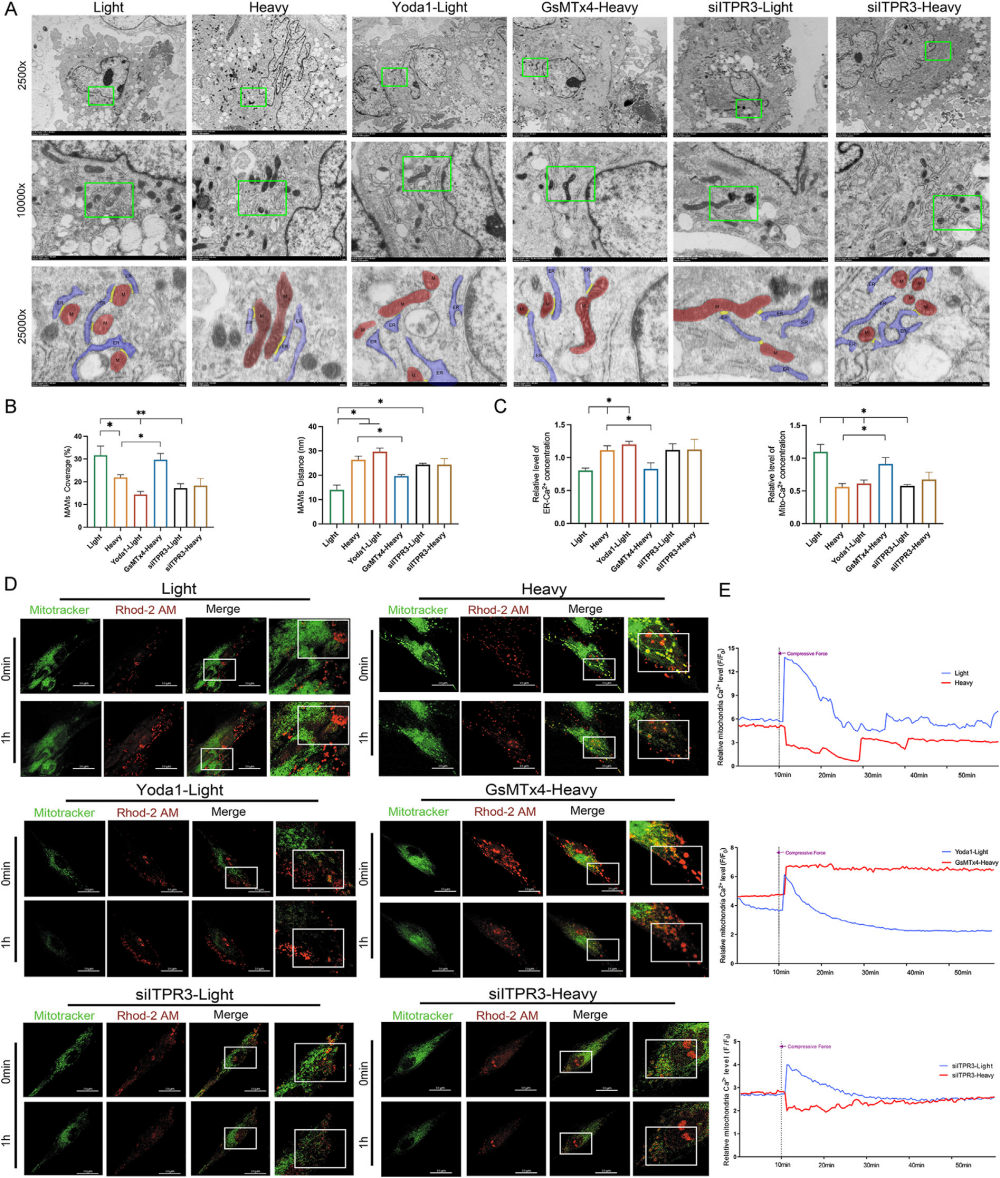
Heavy mechanical force (MF)-activated Piezo1 down-regulates [Ca^2+^]_m_ levels by inhibiting ITPR3 in mitochondria-associated membranes. **(A, B)** Transmission electron microscopy observation and analysis of mitochondria-associated membranes. **(C)** Energy dispersive X-ray analysis of [Ca^2+^]_ER_ and [Ca^2+^]_m_. **(D, E)** Fluorescence observation of [Ca^2+^]_m_ and mitochondria in periodontal ligament stem cells under light or heavy MF stimuli. Scale bar, 10 μm. ITPR3, inositol 1,4,5-trisphosphate receptor type 3.

Calcium transport by MAMs plays a crucial role in immune and inflammatory regulation. Next, we aimed to elucidate the regulatory mechanisms of Piezo1 and ITPR3 in MAMs. Transmission electron microscopy revealed that heavy MF significantly reduced MAM contact compared with light MF (Fig. 3A, B). GsMTx4 significantly promoted MAM contact under heavy MF conditions, whereas Yoda1 significantly suppressed MAM contact under light MF conditions. ITPR3 inhibition significantly decreased MAM contact under light MF conditions.

Energy dispersive X-ray analysis (Fig. 3C) revealed that ER calcium ([Ca^2+^]_ER_) levels were significantly elevated, whereas [Ca^2+^]_m_ levels were significantly lower under heavy MF conditions than under light MF conditions. Yoda1 considerably elevated [Ca^2+^]_ER_ levels and reduced [Ca^2+^]_m_ levels under light MF conditions. GsMTx4 significantly reduced [Ca^2+^]_ER_ levels and elevated [Ca^2+^]_m_ levels under heavy MF conditions. After ITPR3 was inhibited (Fig. S11), [Ca^2+^]_m_ levels significantly decreased under light MF conditions. In addition, Rhod-2 AM was used to label [Ca^2+^]_m_ (Fig. 3D, E; Fig. S12). Compared with light MF, heavy MF significantly reduced [Ca^2+^]_m_ levels. GsMTx4 significantly increased [Ca^2+^]_m_ levels under heavy MF conditions, whereas Yoda1 inhibited [Ca^2+^]_m_ levels under light MF conditions. ITPR3 inhibition significantly decreased [Ca^2+^]_m_ levels, and there was no significant difference between light and heavy MF conditions. These findings suggest that Piezo1 activation under heavy MF conditions hinders the transport of [Ca^2+^]_ER_ to [Ca^2+^]_m_ and that ITPR3 plays a pivotal role in regulating MAMs through Piezo1.

### A low [Ca^2+^]_m_ leads to a decrease in the cytoplasmic release of mtDNA under heavy MF conditions

**Figure 4:**
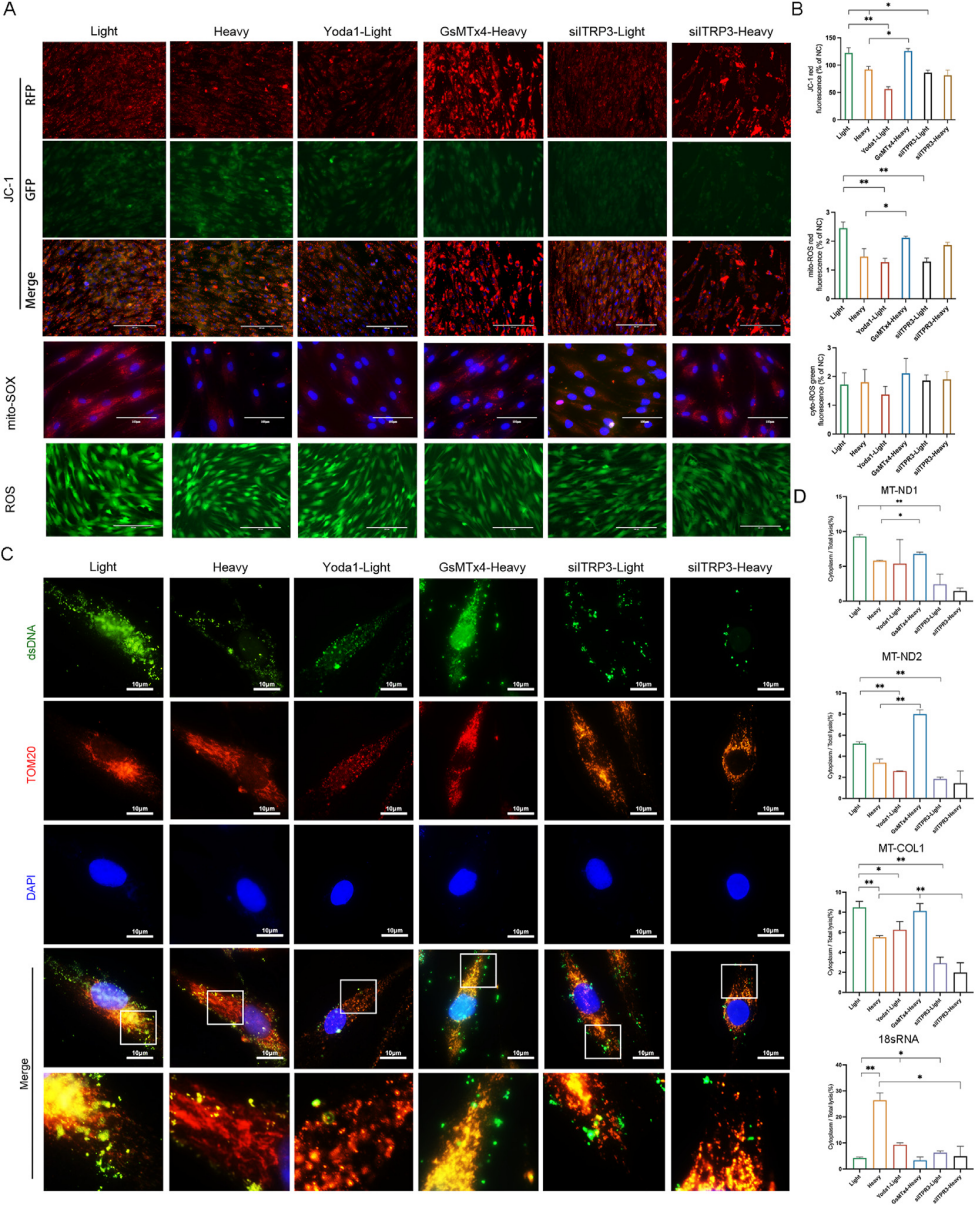
A low [Ca^2+^]_m_ under heavy mechanical force (MF) conditions leads to a decrease in the cytoplasmic release of mitochondrial DNA (mtDNA). **(A, B)** Fluorescence analysis of the mitochondrial membrane potential (Δψm), cytoplasmic reactive oxygen species (ROS), and mitochondrial ROS in each MF group. Scale bar, 200 μm. **(C)** Immunofluorescence analysis of mtDNA. Scale bar, 10 μm. **(D)** Quantitative reverse-transcription PCR analysis of the cytoplasmic ratios of 18sRNA, MT–ND1, MT–ND2, and MT–CO1. MT–ND1/2, mitochondrially encoded NADH dehydrogenase 1/2; MT–CO1, mitochondrially encoded cytochrome C oxidase I.

To elucidate the degree of mitochondrial stress caused by MF stimulation, we initially evaluated the mitochondrial membrane potential (Δψm) (Fig. 4A, B). Yoda1 and ITPR3 inhibition both significantly decreased the Δψm under light MF conditions, whereas GsMTx4 significantly increased the Δψm under heavy MF conditions. Analysis of mitochondrial reactive oxygen species (mito-ROS) levels revealed that compared with that under light MF conditions, Yoda1 and ITPR3 inhibition reduced mito-ROS generation (Fig. 4A, B). Conversely, GsMTx4 significantly increased mito-ROS generation under heavy MF conditions. The intracellular ROS levels did not significantly differ between the groups (Fig. 4A, B).

Immunofluorescence analysis revealed that the mtDNA levels were significantly greater under light MF versus heavy MF conditions. Yoda1 and ITPR3 inhibition significantly reduced mtDNA under light MF conditions, whereas GsMTx4 notably increased mtDNA under heavy MF conditions (Fig. 4C; Fig. S13). Next, we assessed the proportion of cytoplasmic release of mtDNA under light or heavy MF conditions; quantitative reverse-transcription PCR analysis revealed that the proportions of MT–ND1 (mitochondrially encoded NADH dehydrogenase 1), MT–ND2 (mitochondrially encoded NADH dehydrogenase 2), and MT–CO1 (mitochondrially encoded cytochrome C oxidase I) related to mtDNA^23^ significantly increased under light MF conditions compared with those under heavy MF conditions (Fig. 4D). Additionally, Yoda1 and ITPR3 inhibition significantly decreased the proportions of MT–ND1, MT–ND2, and MT–CO1 under light MF conditions, and GsMTx4 significantly increased under heavy MF conditions. These results suggest that a decrease in [Ca^2+^]_m_ under heavy MF conditions leads to the disruption of Δψm and a reduction in mito-ROS, resulting in decreased cytoplasmic mtDNA release.

### cGAS‒STING sensing by the cytoplasmic release of mtDNA plays a regulatory role in OTM

**Figure 5:**
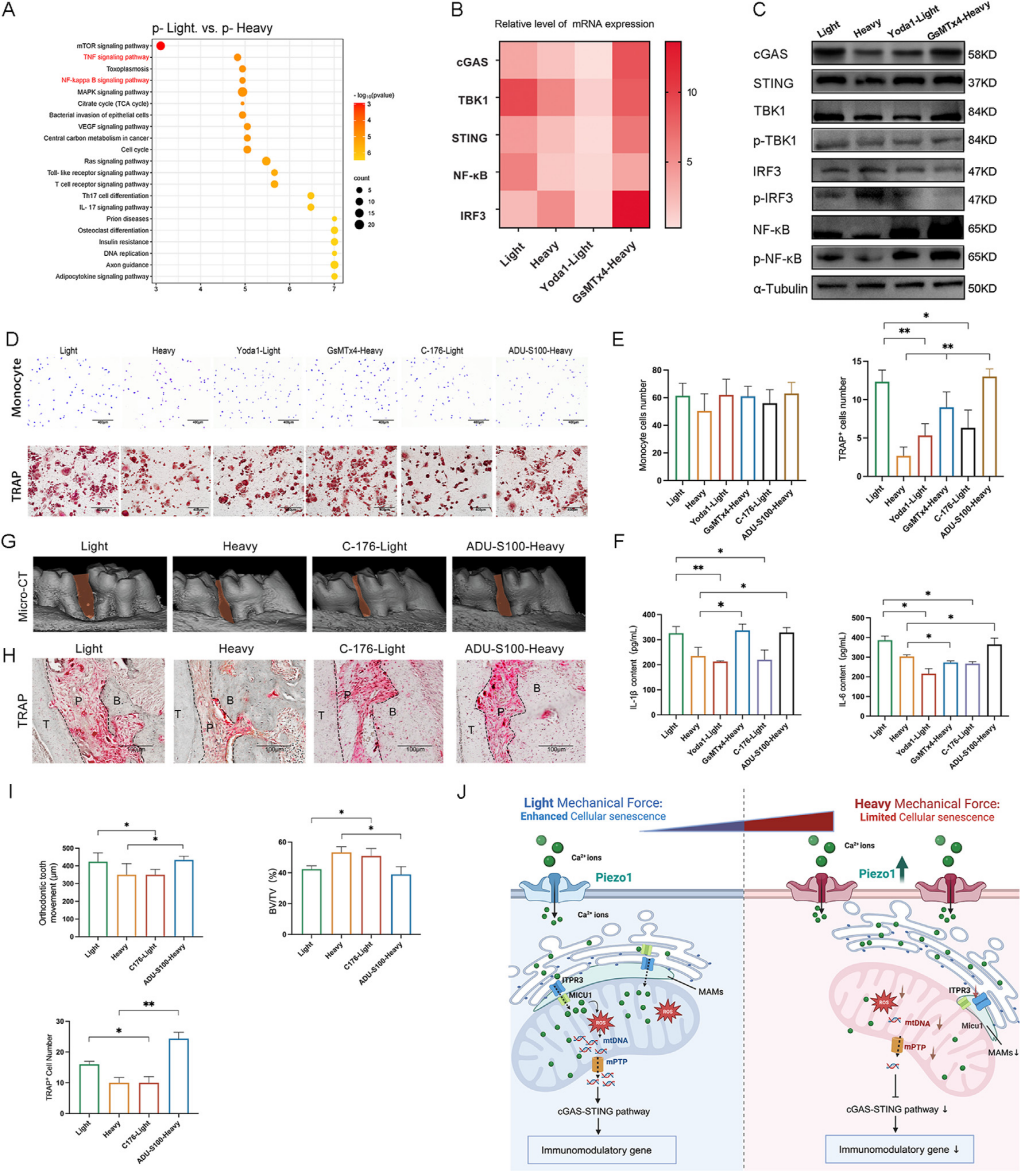
Sensing of the cytoplasmic release of mitochondrial DNA (mtDNA) by cGAS–STING plays a regulatory role in orthodontic tooth movement (OTM). **(A)** Phosphorylation-modified proteome KEGG enrichment analysis. **(B, C)** Quantitative reverse-transcription PCR and Western blot analysis of the cGAS–STING pathway. **(D, E)** Observation and analysis of monocyte chemotactic crystal violet staining and TRAP staining in different supernatants of periodontal ligament stem cells (PDLCs) after mechanical force (MF) stimulation. Scale bar, 400 μm. **(F)** ELISA analysis of IL-1β and IL-6 in the supernatants of PDLCs after MF stimulation. **(G**–**I)** Micro-CT and TRAP staining analysis of orthodontic tooth movement (OTM) model with the STING promoter and inhibitor, respectively. Scale bar, 100 μm. **(J)** Mechanistic diagram of light MF- or heavy MF-induced PDLCs inflammation regulation by Piezo1-mediated [Ca^2+^]_m_ in OTM.

Kyoto Encyclopedia of Genes and Genomes (KEGG) enrichment analysis suggested that the NF-κB (nuclear factor-kappa B) and TNF (tumor necrosis factor) signaling pathways were significantly enriched in the phosphorylation-modified proteome (Fig. 5A). When intracellular mtDNA was depleted with EtBr, a significant decrease in cGAS‒STING and downstream NF-κB was detected under light or heavy MF conditions, indicating that cGAS activation is caused mainly by the cytoplasmic release of mtDNA under MF stress (Fig. S14A, B). The expression of proteins associated with the cGAS‒STING pathway downstream of mtDNA was subsequently examined. Quantitative reverse-transcription PCR and Western blot analyses revealed that the expression of cGAS‒STING pathway-related mRNAs and proteins corresponded with the cytoplasmic release trend of mtDNA under light and heavy MF conditions (Fig. 5B, C; Fig. S14C‒E).

Our results suggested that the supernatants from each group exhibited similar capacities for monocyte recruitment (Fig. 5D, E). TRAP staining results revealed a greater number of TRAP^+^ cells under light MF versus heavy MF conditions (Fig. 5D, E). Additionally, Yoda1 and C-176 significantly decreased the number of TRAP^+^ cells under light MF conditions, whereas GsMTx4 and ADU-S100 significantly increased the number of TRAP^+^ cells under heavy MF conditions. ELISAs revealed that GsMTx4 and ADU-S100 significantly increased the concentrations of IL-1β and IL-6 under heavy MF conditions (Fig. 5F). Yoda1 and C-176 significantly decreased the concentrations of IL-1β and IL-6 under light MF conditions. These findings suggest a potential link between the cGAS‒STING pathway and inflammation, which may be influenced by changes in mtDNA content and mitochondrial dysfunction in response to different MFs.

To examine the effect of the cGAS–STING pathway on the regulation of inflammation by Piezo1 *in vivo*, we performed micro-CT scanning (Fig. 5G–I; Fig. S15) on rat samples. ADU-S100 (a STING agonist) significantly increased the OTM distance and elevated the degree of bone remodeling under heavy MF conditions at 7 days. C-176 (a STING inhibitor) significantly decreased OTM distance and degraded bone remodeling levels under light MF conditions at 7 days. TRAP staining analysis confirmed that the number of TRAP^+^ cells in different MFs followed the above trend (Fig. 5H, I; Fig. S16). These results indicate that STING is crucial in regulating inflammation in OTM.

## Discussion

Our study reveals for the first time that heavy MF-induced Piezo1 decreases [Ca^2+^]_m_ levels by down-regulating ITPR3 expression, ultimately decelerating OTM. We emphasize Piezo1's role in modulating cellular immune responses to MF stimuli and suggest that regulating [Ca^2+^]_m_ levels via Piezo1 could be a promising therapeutic target for accelerating OTM.

Given the important role of PDLCs in the initial perception of mechanical stimuli and the coordination of immune responses related to alveolar bone remodeling, clarifying the altered biological behaviors of PDLCs could elucidate the changes in the rate of OTM under different MFs.^3^^,^^17^ Consistent with the findings of a previous study,^17^ our results confirmed that PDLCs exhibited the strongest osteoclast induction ability under light MF (2 g/cm^2^) conditions and accelerated PDLCs apoptosis under heavy MF (8 g/cm^2^) conditions (Fig. S8, 16). Thus, we designated 2 g/cm^2^ and 8 g/cm^2^ as light and heavy MF conditions, respectively. Whereas various periodontal tissues and cells are affected by MFs during the OTM process, further exploration is needed to determine the precise values of light and heavy MFs that different cells can withstand.

Piezo1, a crucial mechanosensitive ion channel in PDLCs, mediates MF sensing and promotes periodontal homeostasis.^8^^,^^24^ Previous studies have shown that regulating Piezo1 expression affects alveolar bone remodeling during OTM.^8^^,^^25^ Our study demonstrated that heavy MF overactivated Piezo1, slowing OTM and reducing osteoclast activity on the pressure side (Fig. 1). Inhibition of Piezo1 promoted OTM and bone remodeling under heavy MF conditions, indicating that overactivation of Piezo1 under heavy MF conditions is the main reason for slowing alveolar bone remodeling during OTM. A previous study has reported that the orthodontic force with GsMTx4 local injection reduces OTM,^8^ in contrast with our results. We hypothesize that this discrepancy may be due to differences in the magnitude of orthodontic forces and drug intervention dosage applied in the study. Under different MF conditions, Piezo1 activation may influence OTM through various mechanisms. Therefore, to gain a more comprehensive understanding of the role of Piezo1 in OTM, future research should further delineate and investigate the specific ranges of light and heavy orthodontic forces and their effects on OTM.

Ca^2+^ acts as a second messenger to regulate PDLCs physiological and immune responses under MF stress.^26^^,^^27^ Our proteomics results revealed significant differences in calcium ion binding pathways under light and heavy MF conditions (Fig. 2D, E). Interestingly, calcium oscillation analysis revealed that [Ca^2+^]_i_ levels were significantly reduced under heavy MF conditions, whereas Piezo1 inhibition significantly elevated [Ca^2+^]_i_ levels under such conditions (Fig. 2F). Piezo1 activation is thought to allow a greater amount of Ca^2+^ to enter the cell^28^; however, the results of this study revealed the opposite trend in [Ca^2+^]_i_ levels. This effect may be due to the involvement of organelles, such as the ER, in regulating calcium homeostasis in PDLCs by heavy MF-activated Piezo1.^29^^,^^30^ As a crucial calcium pool in PDLCs, the ER maintains calcium homeostasis under MF stimulation.^31^^,^^32^ MAMs bridge Ca^2+^ signaling between the ER and mitochondria, and their key complexes play important roles in the cellular MF stress response.^33^^,^^34^ Our findings confirmed that Piezo1 activation under heavy MF conditions inhibits MAM contact, decreasing [Ca^2+^]_m_ uptake from the ER under heavy MF conditions (Fig. 3A, B). ITPR3 is considered a vital Ca^2+^ transporter protein for regulating MAMs.^35^ Our results demonstrated that ITPR3 was inversely regulated by Piezo1 activation under MF conditions (Fig. 2I; Fig. S9). These factors suggest that reduced contact with MAMs under heavy MF conditions leads to decreased [Ca^2+^]_m_ levels due to ITPR3 regulation.

Decreased [Ca^2+^]_m_ levels impair cellular bioenergetics and lead to organ dysfunction, highlighting the vital role of [Ca^2+^]_m_ under MF conditions in immune regulation.^36^ Previous studies have shown that [Ca^2+^]_m_ stimulates the tricarboxylic acid cycle and oxidative phosphorylation, promoting mito-ROS production in mitochondrial respiratory chain complexes.^36^ Our results also confirmed that the decrease in [Ca^2+^]_m_ levels under heavy MF conditions led to mitochondrial stress, characterized by a disruption of the Δψm and reduced mito-ROS generation (Fig. 4A, B). High [Ca^2+^]_m_ levels increase mitochondrial reactive mito-ROS, which can promote the intracellular release of mtDNA.^37^ Limited mtDNA oxidation by mito-ROS generated under MF stress reportedly activates inflammasome activation^38^^,^^39^; however, long durations and massive mito-ROS and mtDNA generation also reportedly induce cell apoptosis and death.^40^ These different characteristics of mtDNA make it a key factor in activating the cGAS‒STING pathway and producing different cellular outcomes under different stress conditions.^41^^,^^42^ Our findings suggested that the cytoplasmic release of mtDNA was significantly greater under light MF conditions, whereas lower mtDNA and cell apoptosis were detected under heavy MF conditions (Figure 4, Figure 5). Previous studies have suggested that some forms of mtDNA stress can elicit a genotoxic stress sentinel role, preserving nuclear DNA integrity and promoting cell survival and other beneficial adaptive responses, which supports our findings.^38^^,^^39^ Our results revealed that the mtDNA–cGAS–STING pathway is a central mediator of the inflammatory response, thereby contributing to alveolar bone remodeling and tooth movement. Further studies should be conducted to elucidate how cGAS‒STING regulates NF-κB downstream inflammation.

In conclusion, this study demonstrates that heavy MF activates Piezo1, which downregulates [Ca^2+^]_m_ by inhibiting ITPR3 expression, thereby reducing the immune regulatory capacity of PDLCs and decelerating OTM (Fig. 5J). Our findings underscore the pivotal role of Piezo1 in modulating [Ca^2+^]_m_ levels under MF stress, providing new insights into the mechanisms involved in accelerating OTM. These findings pave the way for developing novel clinical strategies to enhance or control OTM. Further research involving larger sample sizes and extended clinical trials is necessary to validate the inflammatory regulatory processes of PDLCs during OTM. Additionally, the broader impacts of heavy orthodontic MF on PDLCs require further investigation to comprehensively understand its potential adverse effects.

## Funding

This project was supported by the 10.13039/100014717Natural Science Foundation of China (No. 82471016, 81470772), Chongqing Talent Program: Innovative Leading Talents (Medical Field, No. CQYC20210303384), Chongqing Medical Scientific Research Project (China) (No. cstc2020jcyj-msxmX0307), and Youth Innovation in Future Medicine (Chongqing Medical University) (No. W0033).

## Author contributions

**Ye Zhu:** Writing – review & editing, Writing – original draft, Investigation, Formal analysis, Data curation. **Xuehuan Meng:** Writing – review & editing, Data curation. **Qiming Zhai:** Writing – review & editing, Writing – original draft, Formal analysis, Conceptualization. **Liangjing Xin:** Writing – review & editing, Writing – original draft, Formal analysis, Conceptualization. **Hao Tan:** Writing – original draft, Investigation. **Xinyi He:** Writing – original draft, Formal analysis. **Xiang Li:** Writing – original draft, Formal analysis. **Guoyin Yang:** Writing – original draft, Data curation. **Jinlin Song:** Writing – review & editing, Writing – original draft, Formal analysis, Conceptualization. **Leilei Zheng:** Writing – review & editing, Writing – original draft, Formal analysis, Conceptualization.

## Conflict of interests

The authors declared no conflict of interests.
