# Supplementary material for: Heavy mechanical force decelerates orthodontic tooth movement via Piezo1-induced mitochondrial calcium down-regulation
Source: Genes Dis. 2024 Sep 15;12(2):101434. doi: 10.1016/j.gendis.2024.101434 (PMC11697055; doi:10.1016/j.gendis.2024.101434)
Supplement: Multimedia component 1 [file mmc1.docx]

**Appendix**

**Appendix Materials & Methods**

**Micro-CT scanning and analysis**

The samples were fixed in a 4% paraformaldehyde solution for 24 h and subsequently scanned via a vivaCT 40 system (Scanco Medical, Switzerland). The samples were scanned at 70 kV and 114 mA with an integration time of 500 ms and a voxel resolution of 10 mm. The distance of OTM was measured by assessing the spacing between the cementum–enamel junction levels of the first and second left molars. In this study, a 200 mm × 200 mm × 600 mm cube of trabecular bone distal to the middle part of the distal buccal root of the maxillary left first molar was selected as the region of interest for analysis. The distance between the cube and the root was 100 mm. The distance and bone volume/total volume (BV/TV) ratio were subsequently calculated on days 1, 3, and 7 following OTM.

**Histological analysis**

Following micro-CT scanning, each sample from the left half of the maxilla was decalcified in a 14% ethylenediaminetetraacetic acid (EDTA) solution at pH 7.4 for two months. All the samples were subsequently dehydrated in a series of alcohol baths and embedded in paraffin. The samples, which included the maxillary molars, were subsequently cut into frontal sections that were 5 mm thick in the sagittal direction.

H&E staining and TRAP staining (Servicebio, China) were performed for the histological analyses. Multinucleated cells adjacent to the tension side of the periodontal region were quantified as TRAP^+^ osteoclasts. Two independent investigators counted the number of TRAP^+^ cells. Primary antibodies were added for immunohistochemistry (IHC) analysis, and the samples were incubated at 4°C overnight. A diaminobenzidine detection kit (ZSGB-BIO, China) was used. Detailed information on the antibodies is provided in Supplementary Table 1.

**CCK8 assay for measuring cell viability**

A Cell Counting Kit 8 (CCK8) assay was used for PDLCs after being subjected to pressures of different force values for 6 hours. PDLCs were plated in 96-well dishes at 3000 cells per well. The cells were cultured in fresh medium for 1 day, and then different static compression MFs were applied to the PDLCs for 6 hours. The MF was removed, and the cells were further cultured. The CCK-8 assay (Beyotime, China) was performed at 1 d, 3 d, and 7 d according to the manufacturer’s protocol.

**Analysis of proteomics using Tandem Mass Tag (TMT) labeling and protein phosphorylation modifications**

Cells cultured in the light force served as the control group. After 6 h mechanical loading time, the PDLCs were analyzed by Tandem Mass Tag (TMT) labeling and protein phosphorylation modifications (n=3). These analyses were conducted by Novogene Co., Ltd. Statistical analysis of protein quantification results using a T-test. Proteins with significant quantitative variances between the experimental and control groups (p<0.05, | log^2^FC |>1.5) were categorized as differentially expressed proteins (DEPs).

**Cellular TRAP staining**

After applying compressive MF, PDLCs were fixed with 4% paraformaldehyde. Tartrate-resistant acid phosphatase (TRAP) staining (Servicebio, China) was performed for the PDLCs according to the instructions.

**Flow cytometry analysis of the cell cycle and apoptosis**

The compressed PDLCs were centrifuged to collect the cell precipitates. Then, 400 µL of ethidium bromide (PI, 50 μg/mL) and 100 µl of RNase A (100 μg/mL) were added, and the mixture was incubated at 4°C in the dark for 30 minutes. Conventional flow cytometry methods were applied, and the outcomes were assessed via the cell cycle fitting software ModFit.

After compressive MFs were applied, cell apoptosis was assessed via an Annexin V-FITC Apoptosis Staining/Detection Kit (Abcam, United Kingdom).

**Calcium oscillation analysis**

Calcium oscillation ([Ca^2+^]_i_) activity was assessed in PDLCs pretreated with Fluo8-AM (10 mM for 15 min at 37°C) following the methodology outlined in a previous study^1^. A glass cylinder was placed over a confluent cell layer, and the magnitudes of compression force were set as 2 g/cm^2^ (light force) and 8 g/cm^2^ (heavy force), as mentioned above. Fluorescence images of the cells were captured via a Leica TCS SP8 confocal microscope. After the PDLCs were observed for 10 min, light and heavy MFs were applied under different conditions via the above method, and observation was continued for 50 min. The scanning rate for 8-bit images 1024 × 1024 pixels in size was 1.12 s per scan. Time-lapse images collected from a single Z plane were recorded at 3-s intervals. The change in the intracellular [Ca^2+^]_i_ is represented by the relative intensity of fluorescence.

**Mitochondrial calcium analysis**

The mitochondrial Ca^2+^ ([Ca^2+^]_m_) levels were quantified in PDLCs pretreated with Rhod-2 AM (5 μM) for 15 min at 37°C, as previously described^2^. The MF application method was the same as mentioned above. Fluorescence images of PDLCs were acquired with a Leica TCS SP8 confocal microscope. The fluorescence activity in the cells before the application of MF was documented by Li J et al. ^3^. The relative intensity of fluorescence represented the change in the intracellular [Ca^2+^]_m_.

**Mitochondrial membrane potential, cytoplasmic ROS, and mito-SOX analysis**

After 1 hour of MF application as described above, a JC-1-mitochondrial membrane potential assay kit was applied following the manufacturer's guidelines. The F(aggregate)/F(monomer) ratio was subsequently assessed. A reactive oxygen species assay kit was used to detect cytoplasmic ROS according to the manufacturer's recommendations. For the detection of cytoplasmic ROS, the excitation wavelength was 488 nm, and the emission wavelength was 525 nm. The MitoSOX Red Assay Kit was used to measure mitochondrial ROS according to the manufacturer's recommendations.

**Transmission electron microscopy and Energy Dispersive X-ray spectroscopy analysis**

After MF treatment, the cultured cells were successively fixed with 2.5% glutaraldehyde for 1 h and 2% osmium tetraoxide for 2 h. After being washed with double distilled water, the cell samples were stained with 0.5% uranyl acetate for 12 h, dehydrated, polymerized, and sectioned into 70–90 nm ultrathin sections. Images were observed and captured via a Tecnai G2 TWIN transmission electron microscope. Mitochondrial perimeter and the proportion of the mitochondrial surface in close association with the endoplasmic reticulum (ER), defined as coverage (<40 nm, ER-mitochondrial contact site distance), were calculated at high resolution (25,000×) for all identified mitochondria^4^. The minimum distance between ER release sites and mitochondrial Ca²⁺ uptake sites, designated as MAMs (mitochondria-associated membranes) distance, was measured using straight-line measurements. Three random points from the ER to the nearest outer mitochondrial membrane were selected and measured. All image analyses were performed using ImageJ software. Energy dispersive X-ray spectroscopy (EDX) was performed as previously described^5^. Data on oxygen (O), carbon (C), lead (Pb), and calcium (Ca) were collected from the TEM–EDX analysis. C, O, and Pb were used as controls.

**Immunofluorescence analysis**

After treatment, the cells were fixed in a 4% paraformaldehyde solution for 15 min. The cells were blocked for 2 h at 4°C. Primary antibodies were diluted in a blocking solution and incubated for 18 h at 4°C. The cells were washed with PBS containing 0.1% Triton X-100 and exposed to secondary antibodies diluted in blocking solution at 22°C for 1 h in the dark. Following multiple washes, the coverslips were affixed to a microscope slide with an antifade reagent. The details of the antibodies used can be found in Supplementary Table 1. Immunofluorescence analysis of mitochondrial DNA was performed as previously described^6^.

**Western blot analysis**

Frozen cells were lysed with radio immunoprecipitation assay (RIPA) buffer for 15 minutes, followed by centrifugation to isolate the proteins. Proteins were separated by 8–12% SDS‒PAGE, followed by transfer, blocking, washing, and incubation with specific primary and secondary antibodies. Comprehensive details can be found in Supplementary Table 1. The reagents used for western blotting are presented in Supplementary Table 1. An image lab was used to detect and visualize the protein bands, and alpha-Tubulin was used for normalization.

**Quantitative reverse transcription-polymerase chain reaction (qRT-PCR)**

Cellular RNA was extracted via TRIzol reagent (Invitrogen, United States), and RNA was reverse transcribed to cDNA. RT‒qPCR was conducted to quantitatively analyze the DNA content. The expression levels were normalized against that of ACTB. The set of deltaCq replicates for the control and tested samples, normalized against the geometric means of the reference genes, was used for statistical testing and estimation of the P values. The primers used for qRT‒PCR are presented in Supplementary Table 3.

**Measurement of cytosolic mtDNA proportion**

After different MFs were applied to PDLCs, cytosolic MT-ND1, MT-ND2, MT-COL1, and 18s RNA levels were analyzed via general PCR or RT‒qPCR ^7-9^. Half of the cells were lysed via mild lysis buffer, while the remaining half were lysed via strong lysis buffer. Cellular cytoplasm was lysed with 0.1% NP-40 for 20 min on ice, followed by centrifugation at 14,000×g for 20 min at 4°C. Cytosolic MT-ND1, MT-ND2, MT-COL1 and 18sRNA from the supernatant cytosolic fraction and total MT-ND1, MT-ND2, MT-COL1 and from the total lysate were isolated via a TIANamp Genomic DNA Kit. General PCR, followed by agar gelatin electrophoresis and RT‒qPCR, as detailed previously, was employed for DNA analysis, with normalization to the levels of MT-ND1, MT-ND2, MT-COL1, and 18sRNA in the total lysate.

**siRNA transfection**

For the inhibition of ITPR3, Lipofectamine 2000 (Invitrogen, United States) was used to transfect NP cells with 100 nM ITPR3 small-interfering RNA (si-ITPR3) or the control for 72 hours, followed by immediate exposure to 100 μM TBHP. The siRNA sequences are provided in Supplementary Table 2. Western blotting and qRT‒PCR were used to confirm the knockdown of ITPR3 in PDLCs.

(Appendix Fig.9).

**Depletion of mtDNA by EtBr**

The PDLCs were kept in α-MEM supplemented with ethidium bromide (EtBr) at 1 μg/ml to deplete mtDNA. After 48 h, the EtBr medium was removed, the cells were washed with 2 mL of PBS, and α-MEM was added.

**Enzyme-linked immunosorbent assay**

The supernatants of the culture medium after different MF loadings were added to PDLCs were quantified via commercially available duo-set ELISA kits for human IL-1β (DLB50) and IL-6 (D6050B). The ELISA analysis was conducted following the protocol for the ELISA kits.

**THP-1 cell-derived macrophage Transwell assay**

The supernatants were collected from treated PDLCs. THP-1 cells were added to the upper chamber at 1 × 10^5^ cells/well with phorbol myristate acetate (PMA, 100 ng/mL). The supernatant was then added to the lower chamber. The cells were incubated for 24 hr. The cells stained with crystal violet on the bottom surface were considered migrated cells. For the crystal violet assay, the cells were washed with PBS, fixed for 15 min in 3.7% formaldehyde, and stained with crystal violet.

**THP-1 cell-derived osteoclastogenesis**

THP-1 cells were seeded at 1 × 10^5^ cells per well in 24-well plates and treated with PMA (100 ng/mL) for 48 h. Subsequently, the culture medium was changed to PDLC-derived supernatants supplemented with M-CSF (20 ng/mL) and RANKL (20 ng/mL). The supernatants were replaced, and the cellular morphology was monitored at 2-day intervals over 12 days. TRAP staining was subsequently conducted.

**Statistical analysis**

Statistical analysis was conducted using the SPSS 22.0 Software package (IBM Corporation, Armonk, NY, USA). Data were presented as line graphs with mean values ± SD. Statistical comparison was performed using two-tailed Student’s t test or one‐way analysis of variance (ANOVA) with Tukey’s post hoc test. p < 0.05 was considered statistically significant. All experiments were performed at least in triplicate and repeated 3 times.

**Appendix References**

1. Ei Hsu Hlaing E, Ishihara Y, Wang Z, Odagaki N, Kamioka H. Role of intracellular Ca(2+)-based mechanotransduction of human periodontal ligament fibroblasts. *Faseb j*. Sep 2019;33(9):10409-10424. doi:10.1096/fj.201900484R

2. Chang Y, Wang C, Zhu J, et al. SIRT3 ameliorates diabetes-associated cognitive dysfunction via regulating mitochondria-associated ER membranes. *J Transl Med*. Jul 22 2023;21(1):494. doi:10.1186/s12967-023-04246-9

3. Li J, Qi F, Su H, et al. GRP75-faciliated Mitochondria-associated ER Membrane (MAM) Integrity controls Cisplatin-resistance in Ovarian Cancer Patients. *Int J Biol Sci*. 2022;18(7):2914-2931. doi:10.7150/ijbs.71571

4. Liu Y, Ma X, Fujioka H, Liu J, Chen S, Zhu X. DJ-1 regulates the integrity and function of ER-mitochondria association through interaction with IP3R3-Grp75-VDAC1. *Proc Natl Acad Sci U S A*. Dec 10 2019;116(50):25322-25328. doi:10.1073/pnas.1906565116

5. Hernansanz-Agustín P, Choya-Foces C, Carregal-Romero S, et al. Na(+) controls hypoxic signalling by the mitochondrial respiratory chain. *Nature*. Oct 2020;586(7828):287-291. doi:10.1038/s41586-020-2551-y

6. Victorelli S, Salmonowicz H, Chapman J, et al. Apoptotic stress causes mtDNA release during senescence and drives the SASP. *Nature*. Oct 2023;622(7983):627-636. doi:10.1038/s41586-023-06621-4

7. Yu CH, Davidson S, Harapas CR, et al. TDP-43 Triggers Mitochondrial DNA Release via mPTP to Activate cGAS/STING in ALS. *Cell*. Oct 29 2020;183(3):636-649.e18. doi:10.1016/j.cell.2020.09.020

8. Chung KW, Dhillon P, Huang S, et al. Mitochondrial Damage and Activation of the STING Pathway Lead to Renal Inflammation and Fibrosis. *Cell Metab*. Oct 1 2019;30(4):784-799.e5. doi:10.1016/j.cmet.2019.08.003

9. Zhang W, Li G, Luo R, et al. Cytosolic escape of mitochondrial DNA triggers cGAS-STING-NLRP3 axis-dependent nucleus pulposus cell pyroptosis. *Exp Mol Med*. Feb 2022;54(2):129-142. doi:10.1038/s12276-022-00729-9

**Appendix Figure**


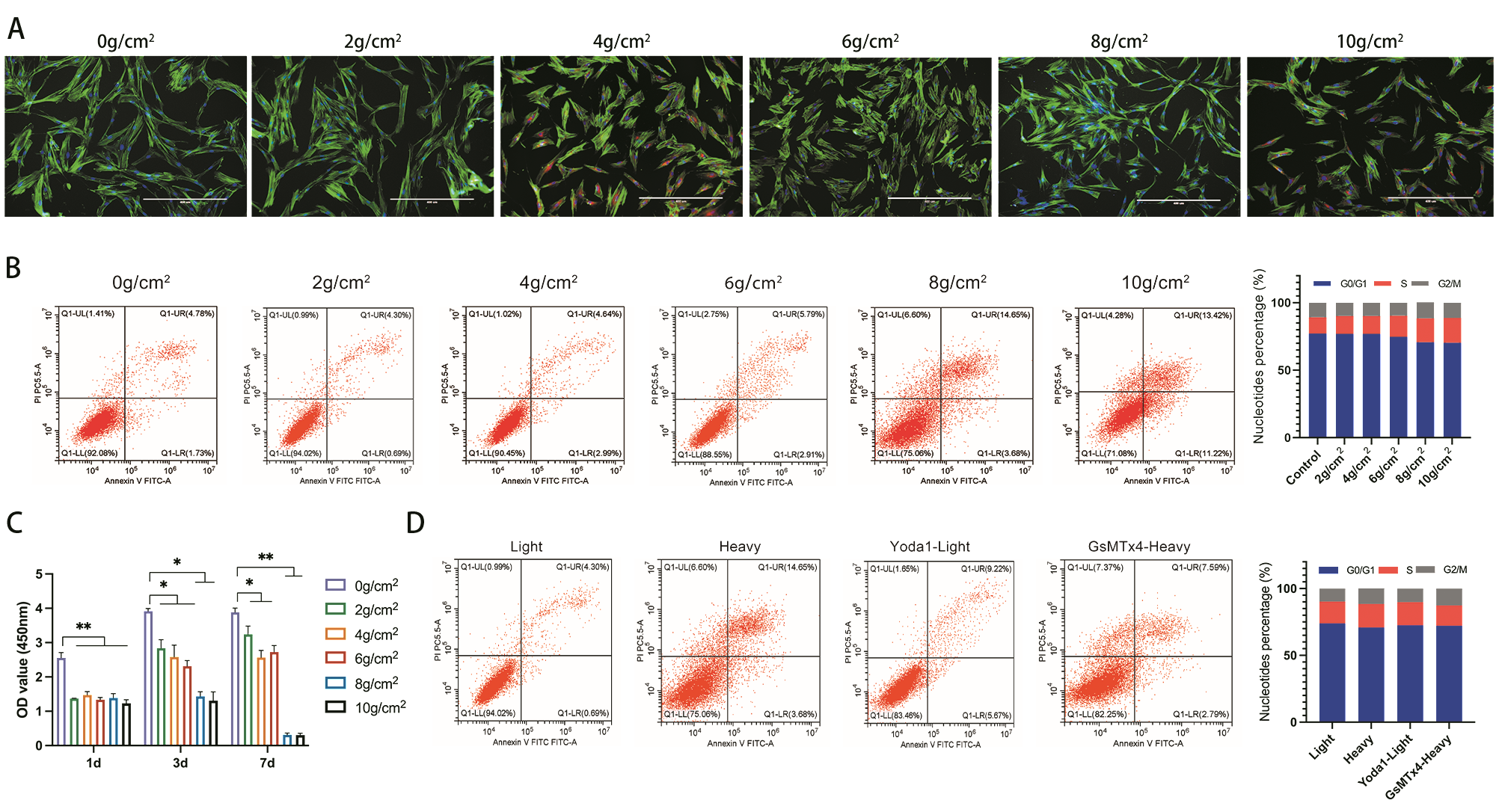


**Figure S1.** Comparison of the effects of light and heavy MFs on PDLCs in vitro. **(A)** Observation of PDL cellular cytoskeleton fluorescence. **(B)** Apoptosis and cell cycle detection of PDLCs cultured with 0 g/cm^2^, 2 g/cm^2^, 4 g/cm^2^, 6 g/cm^2^, 8 g/cm^2^ and 10 g/cm^2^ MFs. **(C)** CCK8 analysis was performed to examine the proliferation of PDLCs after MF stress on days 1, 3, and 7 (N=3; Error bars, mean ± SD, * P<0.05, ** P<0.01). **(D)** Apoptosis and cell cycle detection of PDLCs treated with Piezo1 agonists (Yoda1) and inhibitors (GsMTX4).


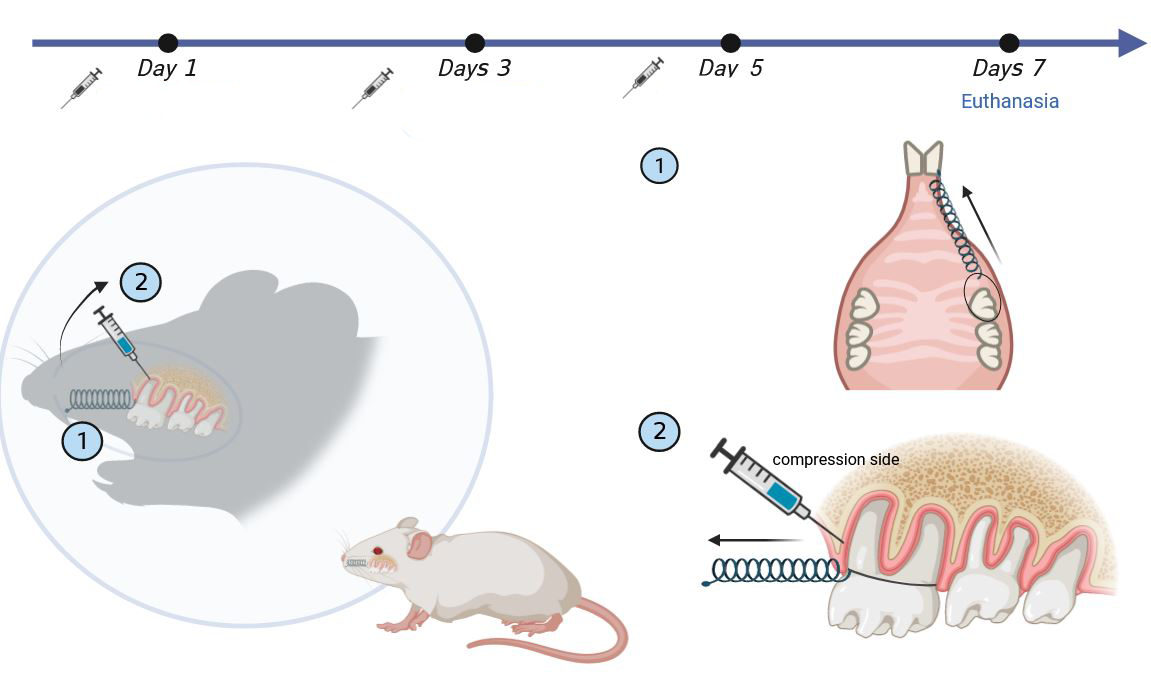


**Figure S2.** OTM model schematic diagram on days 1, 3, and 7 in Fig. 1.


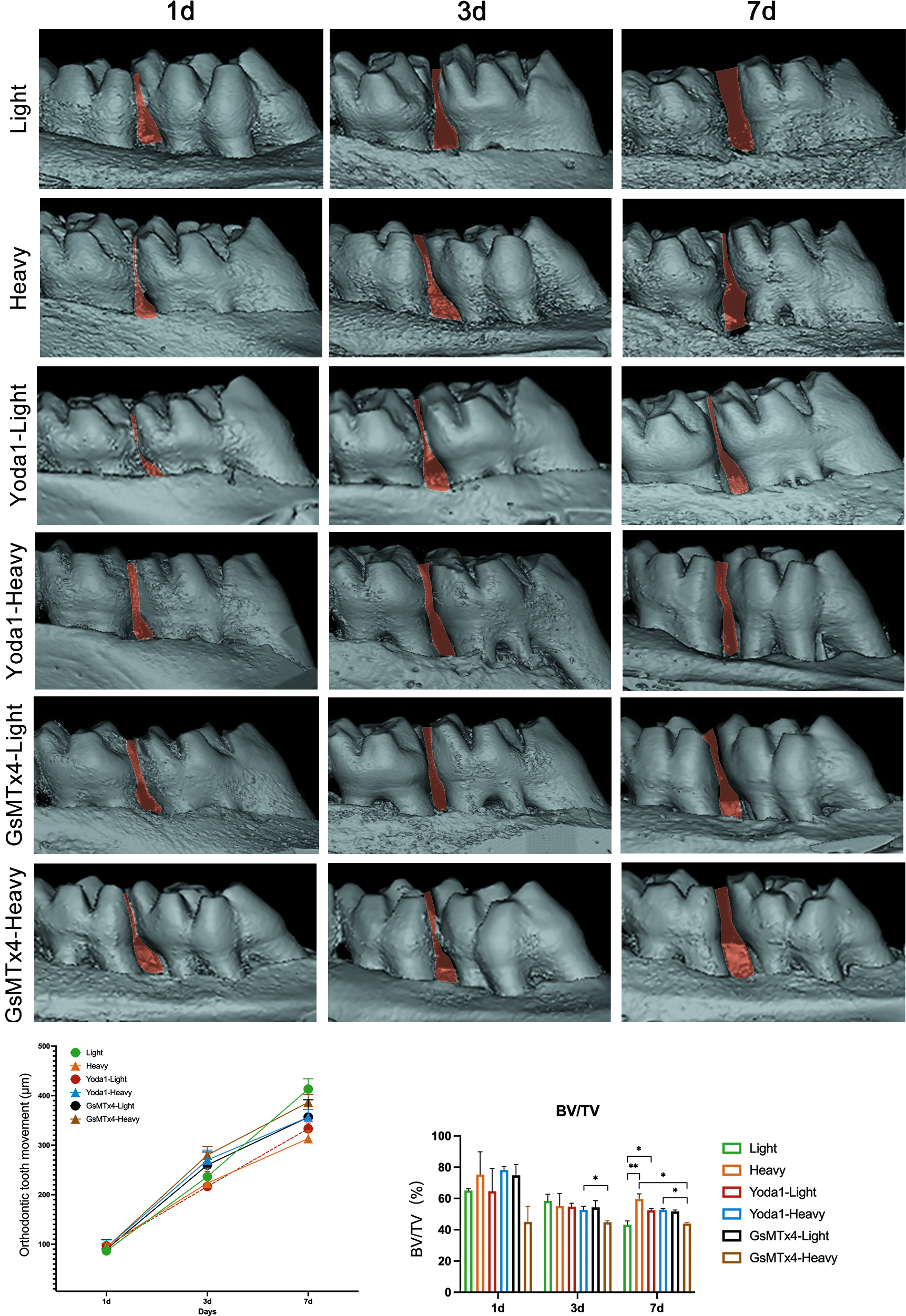


**Figure S3.** Micro-CT analysis of maxillary orthodontic tooth movement on days 1, 3, and 7 after intervention with Piezo1 agonists (Yoda1) and inhibitors (GsMTX4) is shown in Fig. 1 A. N=3; Error bars, mean ± SD, * P<0.05, * * P<0.01.


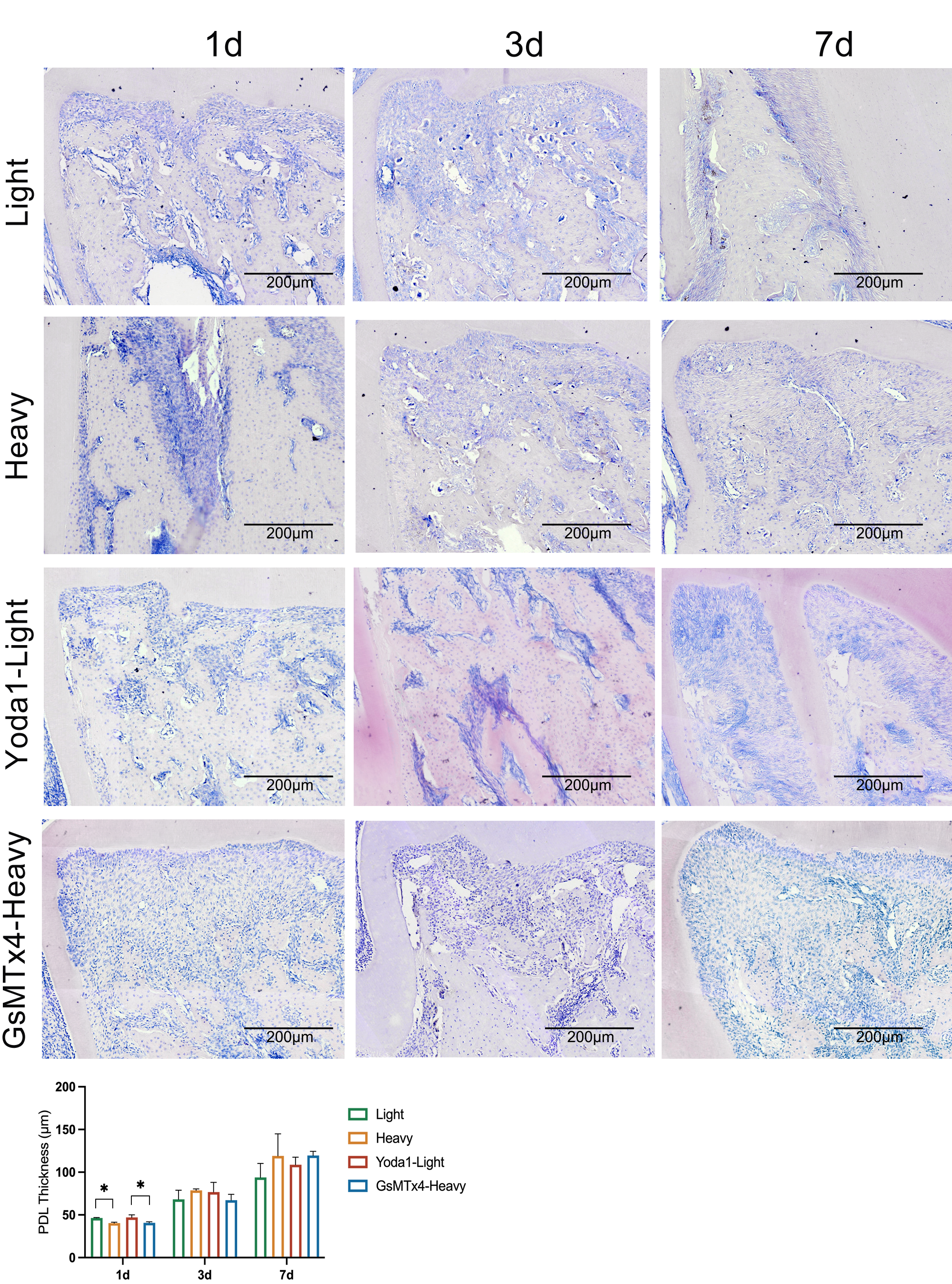


**Figure S4.** Hematoxylin‒eosin staining and analysis of orthodontic tooth roots on days 1, 3, and 7 after intervention with Piezo1 agonists (Yoda1) or inhibitors (GsMTX4) are shown in Fig. 1C (scale bar: 200 μm). N=3; Error bars, mean ± SD, * P<0.05.

**
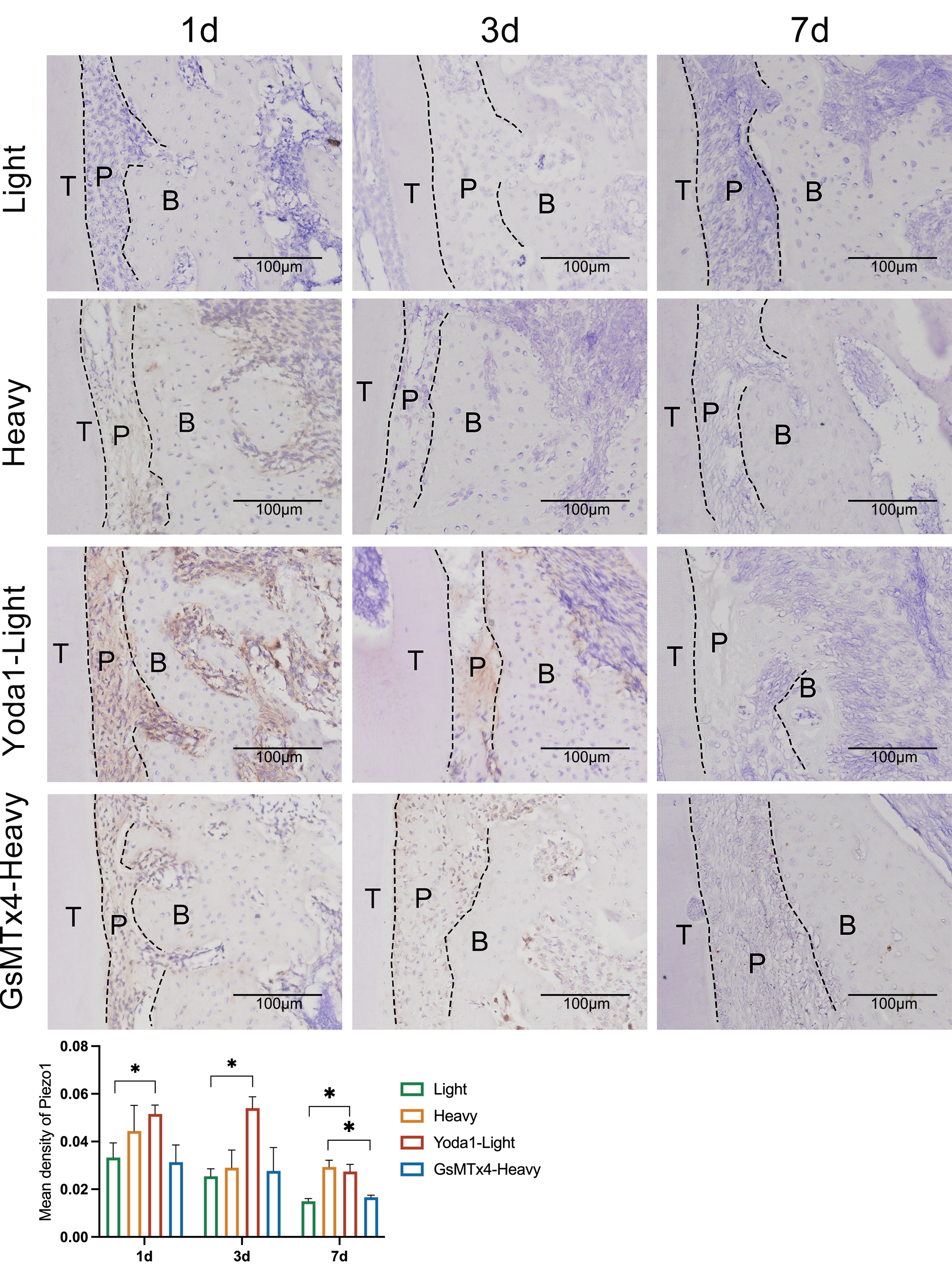
**

**Figure S5.** Immunohistochemical analysis of Piezo1 was conducted on days 1, 3, and 7 after treatment with Piezo1 agonists (Yoda1) or inhibitors (GsMTX4), as shown in Fig. 1E (scale bar: 100 μm). N=3; Error bars, mean ± SD, * P<0.05.


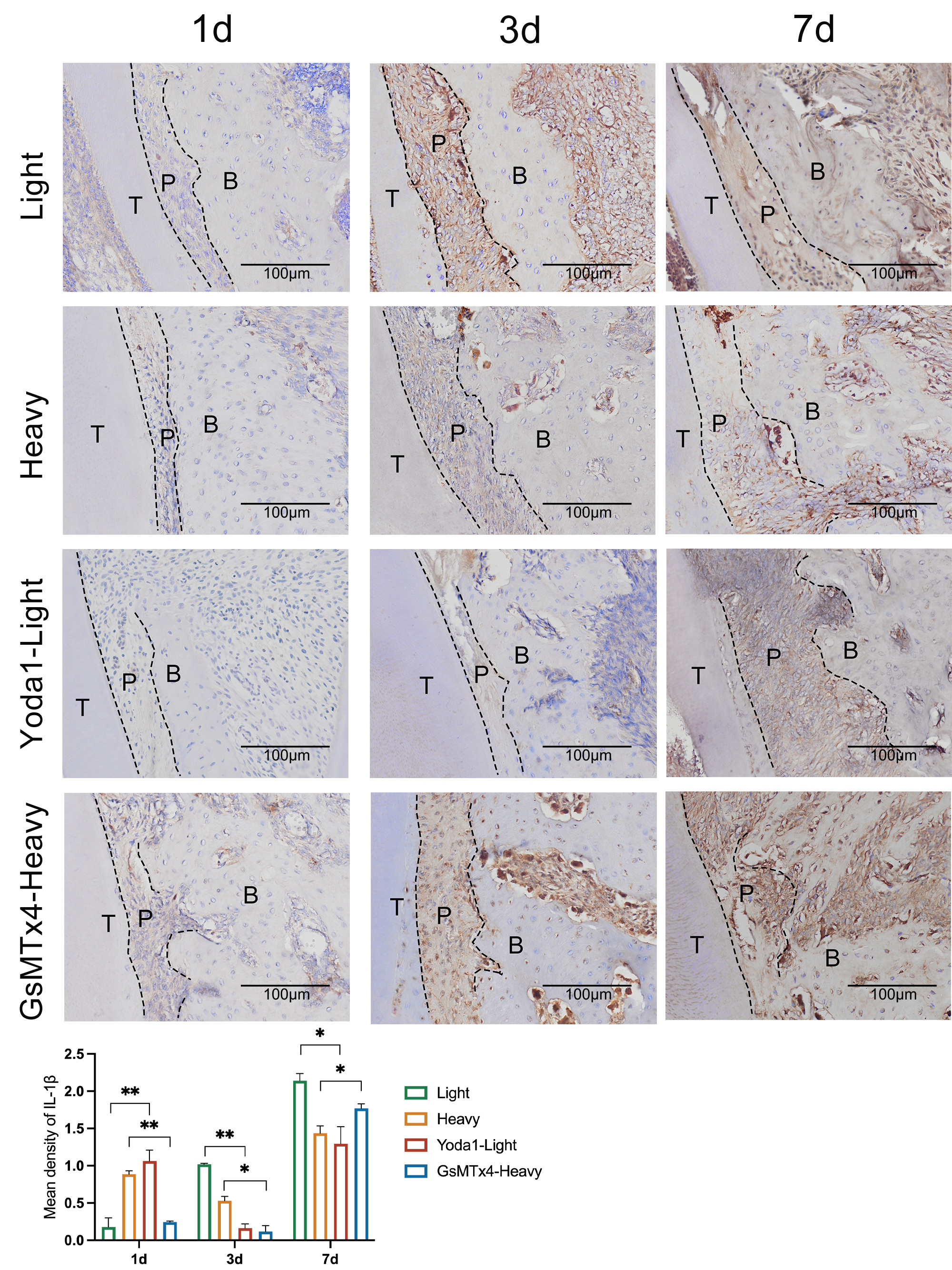


**Figure S6.** Immunohistochemical analysis of IL-1β was conducted on days 1, 3, and 7 after treatment with Piezo1 agonists (Yoda1) or inhibitors (GsMTX4), as shown in Fig. 1G (scale bar: 100 μm). N=3; Error bars, mean ± SD, * P<0.05, ** P<0.01.


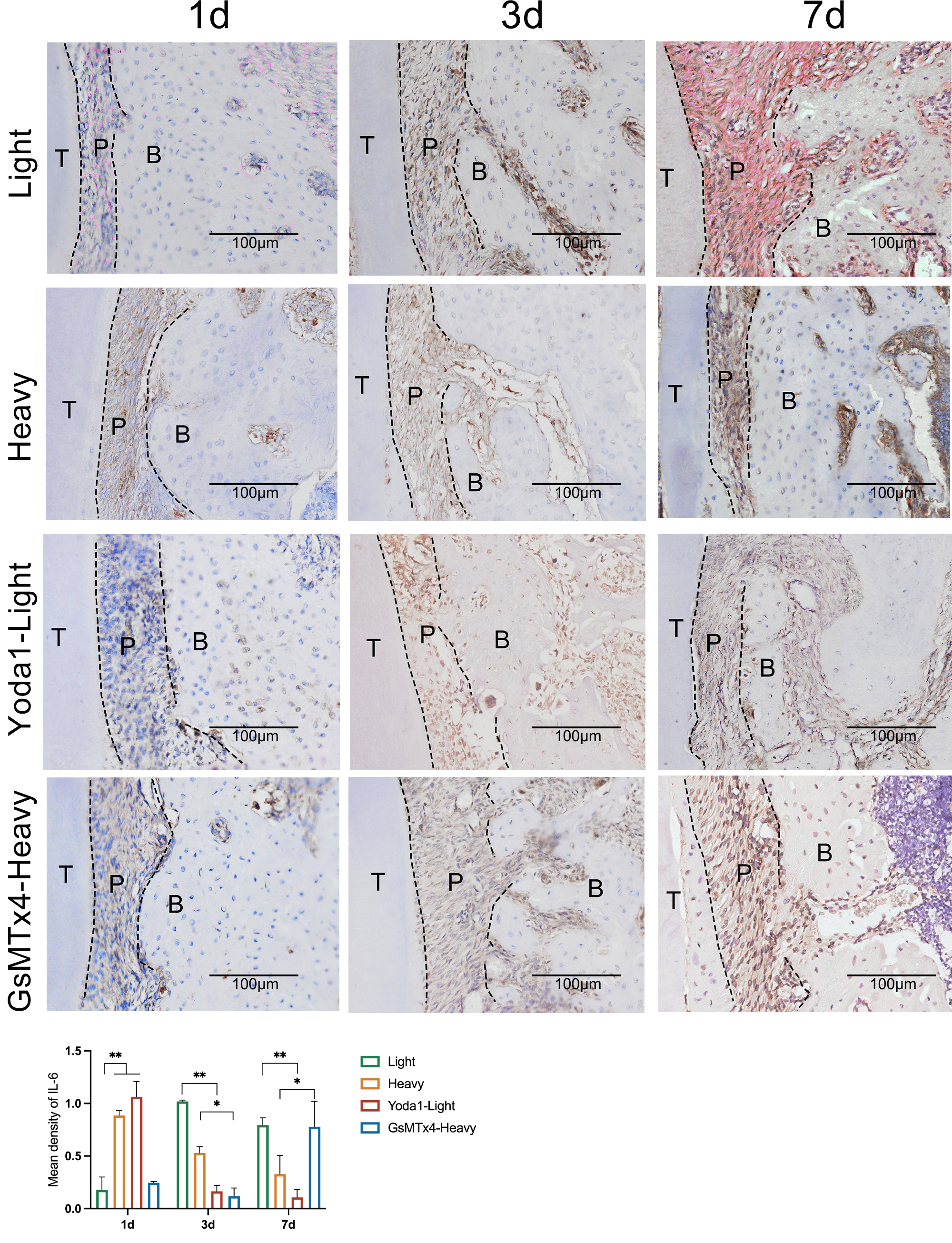


**Figure S7.** Immunohistochemical analysis of IL-6 was conducted on days 1, 3, and 7 after treatment with Piezo1 agonists (Yoda1) or inhibitors (GsMTX4), as shown in Fig. 1G (scale bar: 100 μm).N=3; Error bars, mean ± SD, * P<0.05, ** P<0.01.


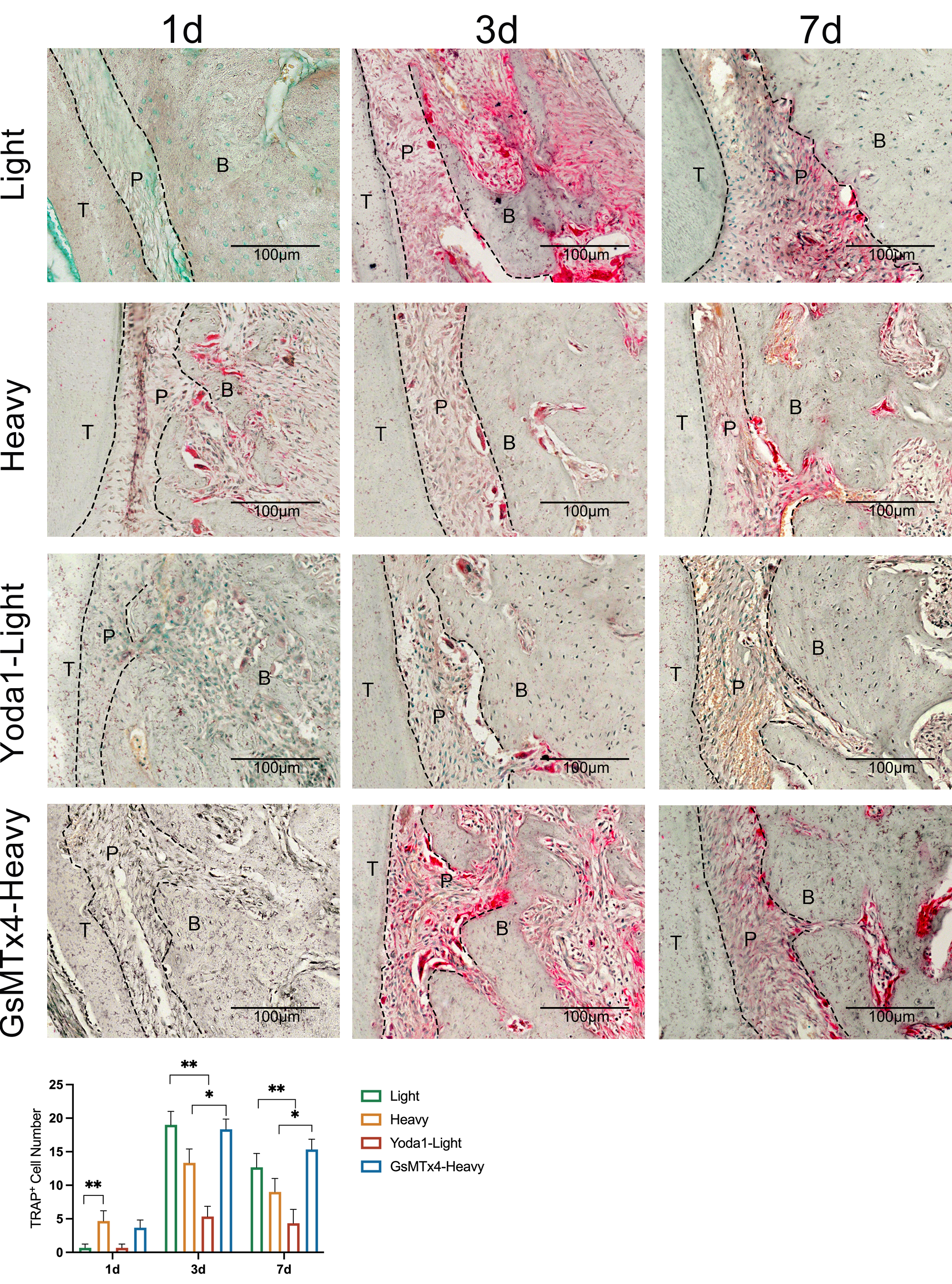


**Figure S8.** Tartrate-resistant acid phosphatase staining and analysis of orthodontic tooth roots on days 1, 3, and 7 after intervention with Piezo1 agonists (Yoda1) and inhibitors (GsMTX4) are shown in Fig. 1I (scale bar: 100 μm). N=3; Error bars, mean ± SD, * P<0.05,** P<0.01.


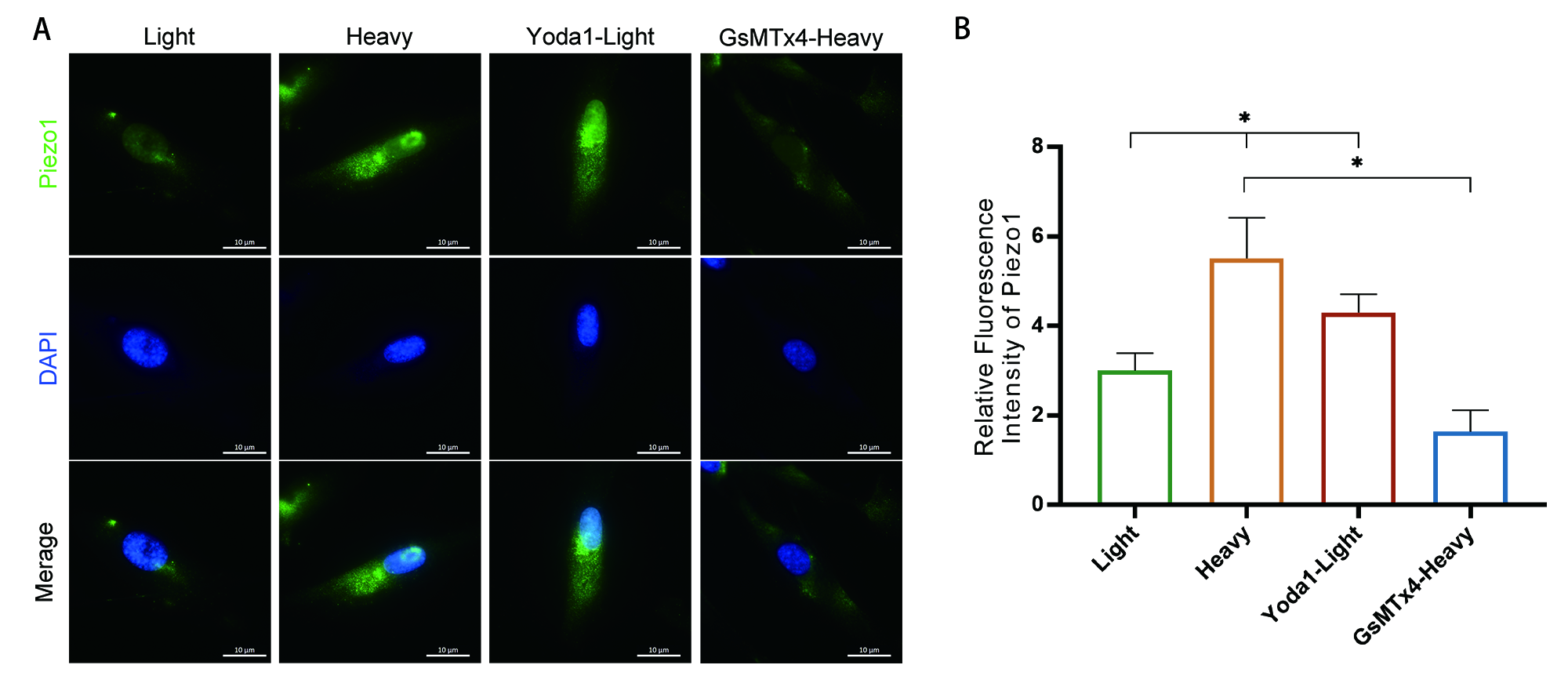


**Figure S9.** Immunofluorescence observation **(A)** and analysis **(B)** of Piezo1 expression in PDLCs loaded with MFs.


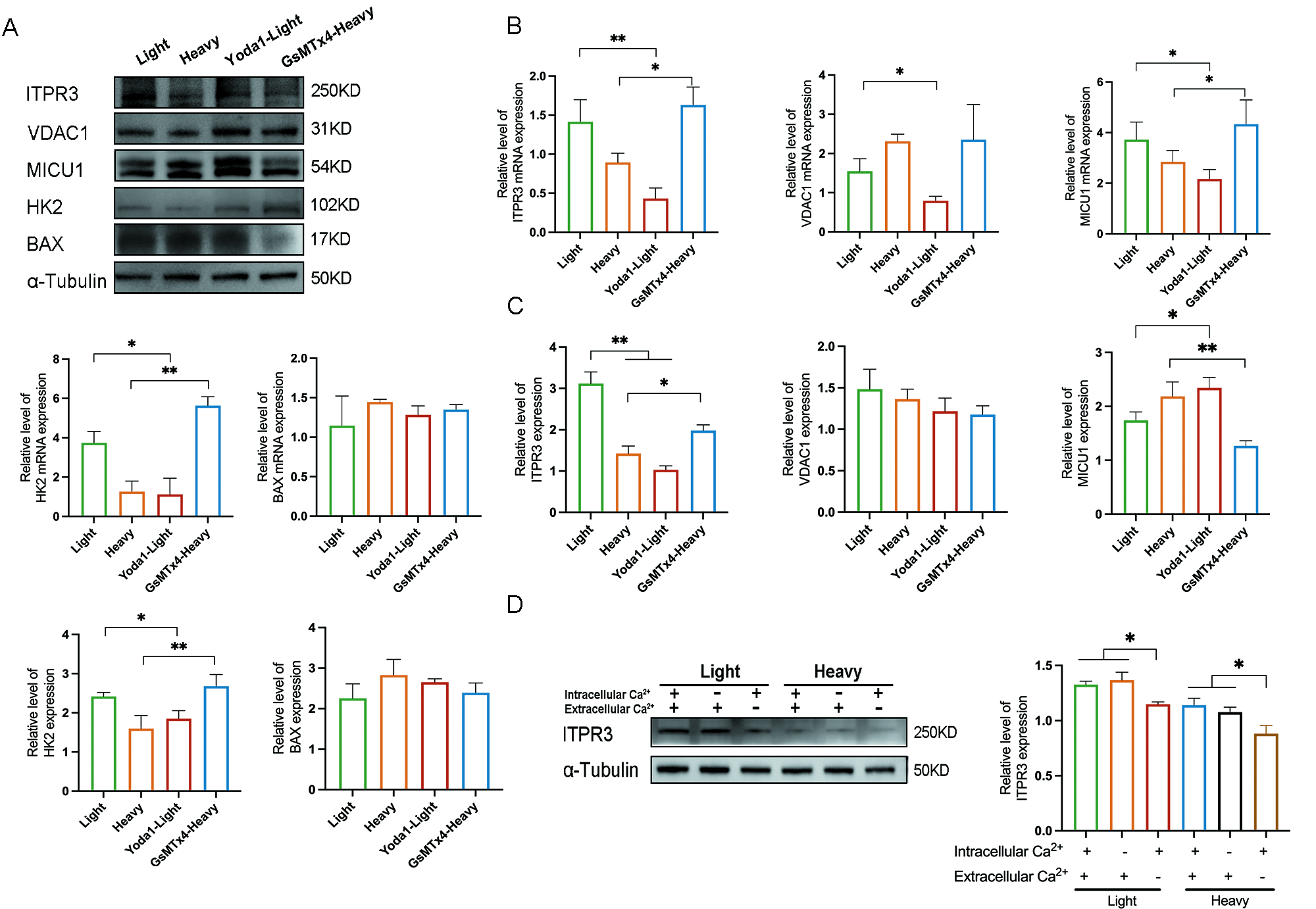


**Figure S10. (A, B, and C)** A western blot of MAM-related calcium signaling protein expression is shown in Figure 2I. **(D)** Quantitative analysis of the protein expression of ITPR3 in culture media with different calcium concentrations. N=3; Error bars, mean ± SD, * P<0.05, ** P<0.01.


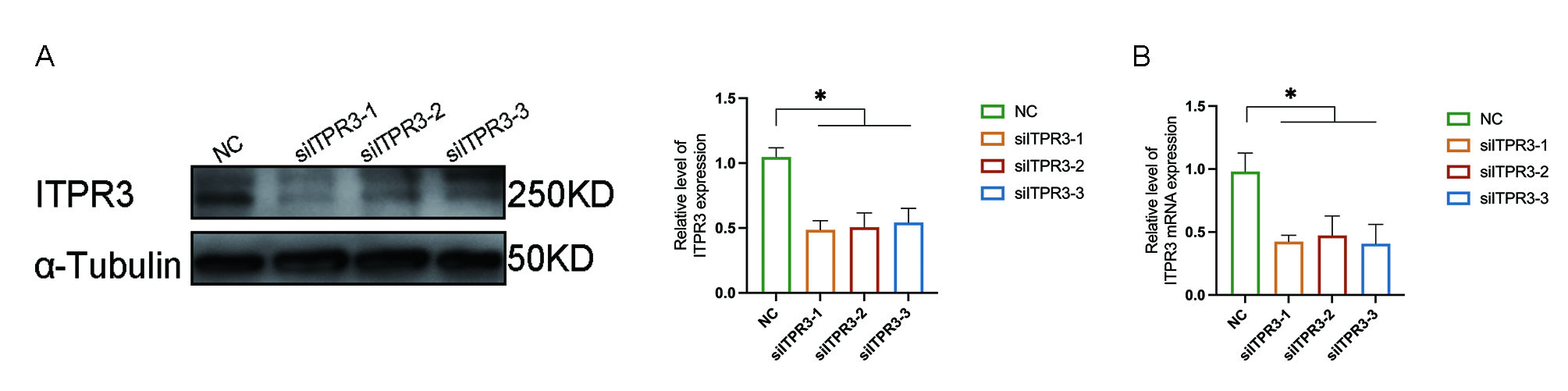


**Figure S11. (A and B)** Western blotting confirmed the successful knockdown of ITPR3 in PDLCs. **(C)** Quantitative RT‒PCR confirmed the successful knockdown of ITPR3 in PDLCs. N=3; Error bars, mean ± SD, * P<0.05.


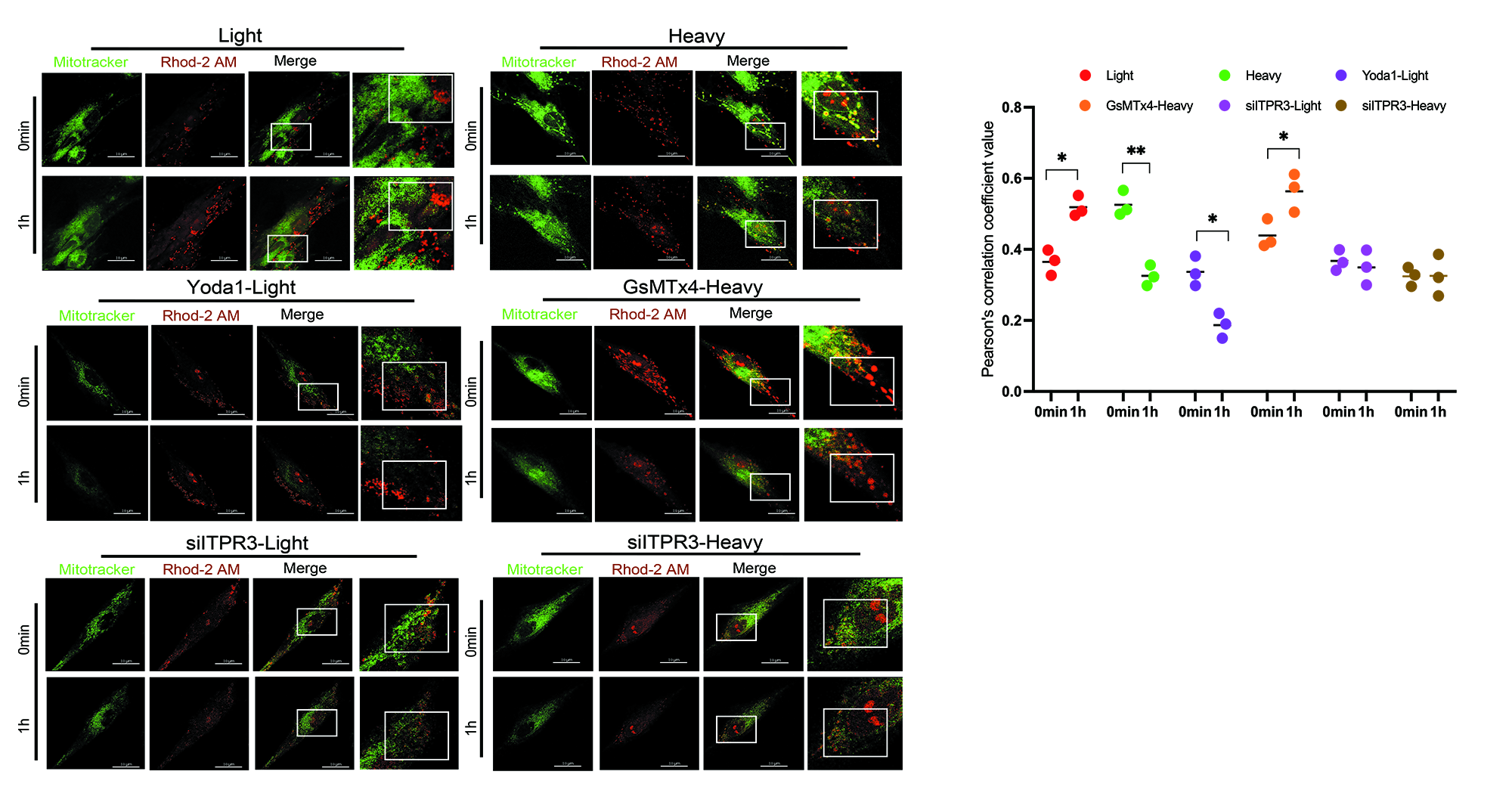


**Figure S12.** The immunofluorescence colocalization analysis of [Ca^2+^]_m_ (Red) and mitochondria (Green) is shown in Fig. 4 C. N=3; Error bars, mean ± SD, * P<0.05, ** P<0.01.


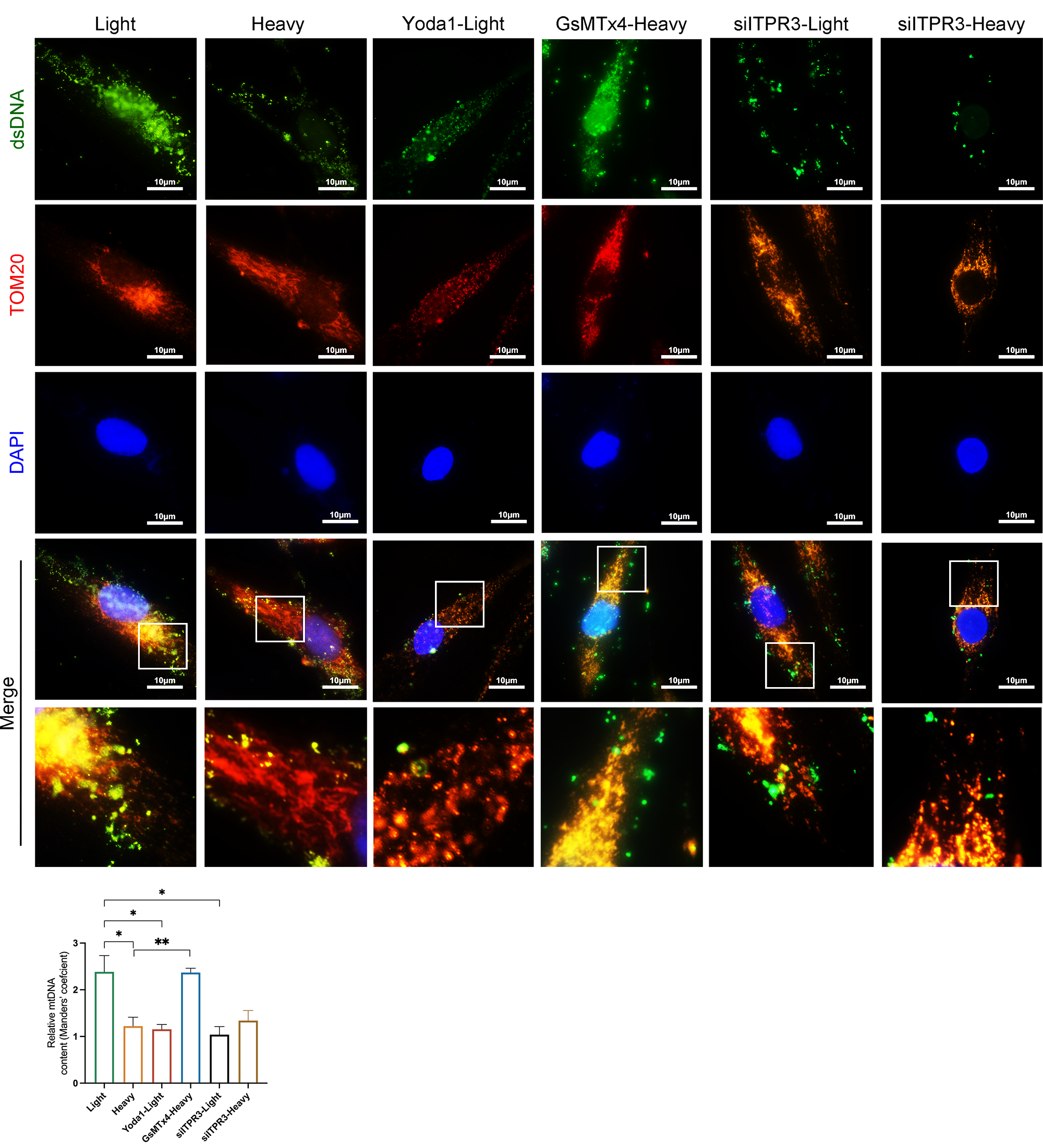


**Figure S13.** The immunofluorescence colocalization analysis of dsDNA and TOM20 is shown in Fig. 4 C. N=3; Error bars, mean ± SD, * P<0.05, ** P<0.01.


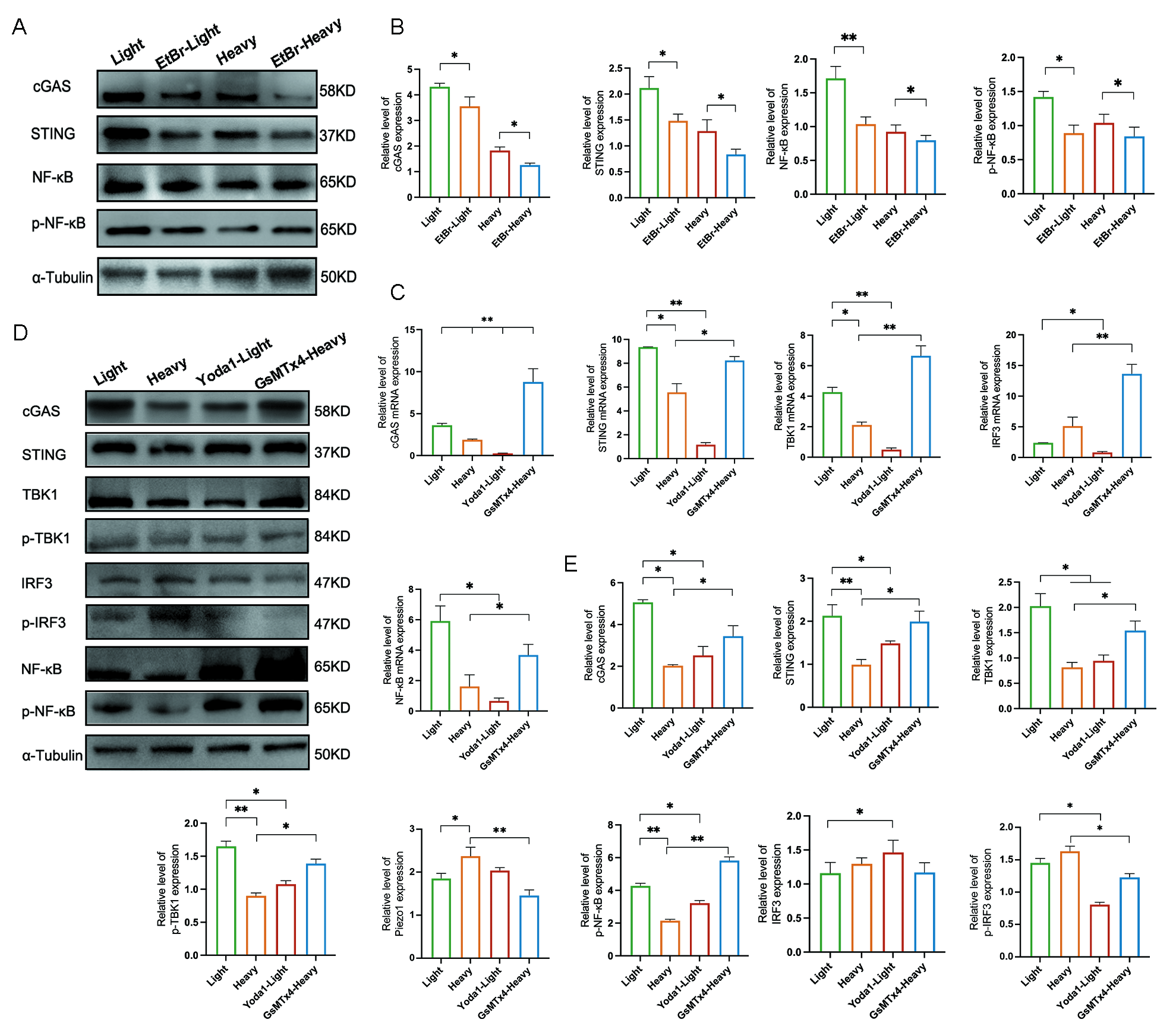


**Figure S14. (A and B)** Quantitative western blot analysis of cGAS‒STING pathway-related protein expression under EtBr intervention. **(C, D, and E)** Quantitative qRT‒PCR and western blot analysis of cGAS-STING pathway-related protein expression under Piezo1 agonist (Yoda1) and inhibitor (GsMTX4) intervention, as shown in Figure 5 C. N=3; Error bars, mean ± SD, * P<0.05, ** P<0.01.


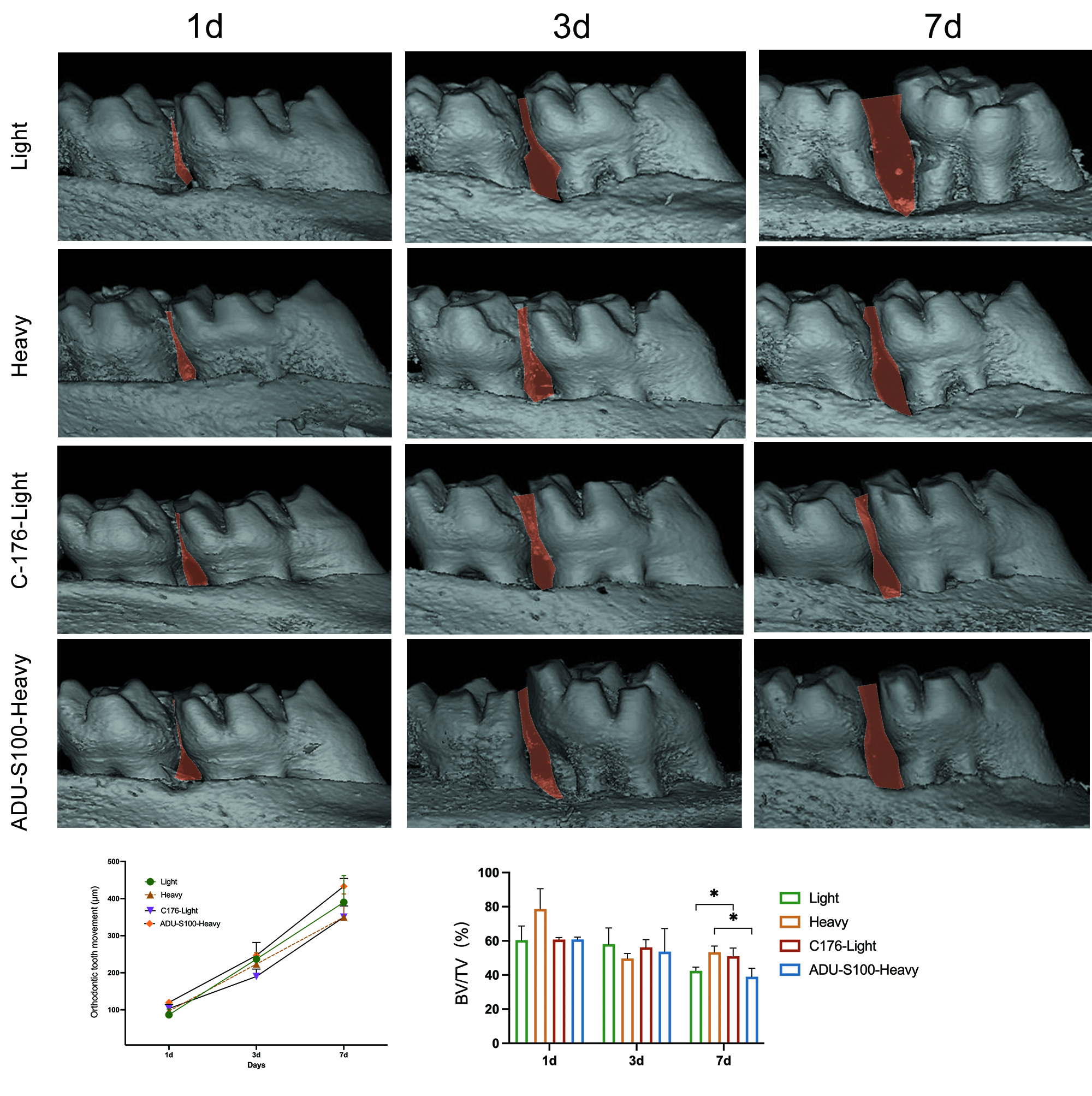


**Figure S15.** Micro-CT analysis of maxillary orthodontic tooth movement on days 1, 3, and 7 after intervention with a STING agonist (ADU-S100) or inhibitor (C-176) is shown in Fig. 5G. N=3; Error bars, mean ± SD, * P<0.05, * * P<0.01.


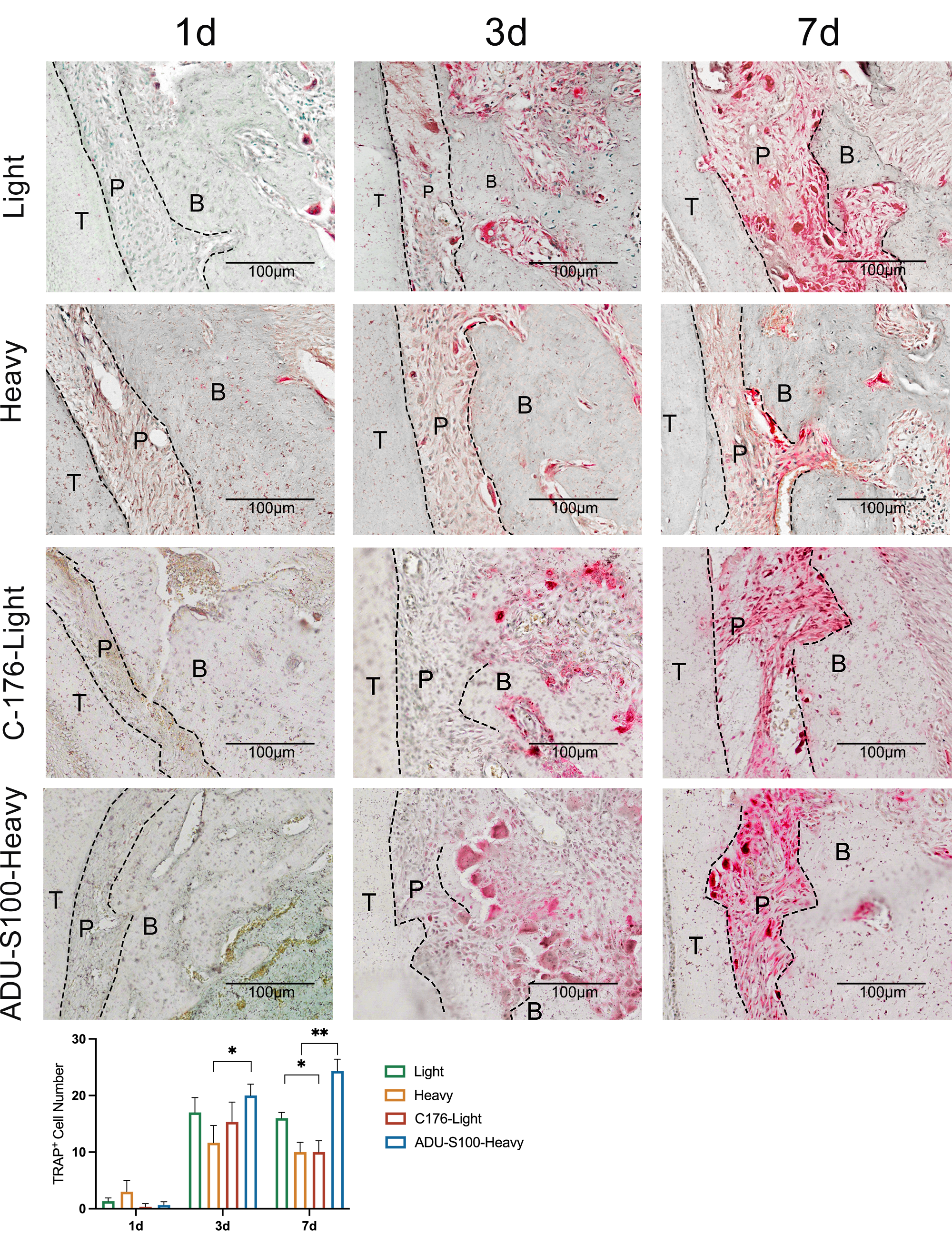


**Figure S16.** Tartrate-resistant acid phosphatase staining and analysis of orthodontic tooth roots on days 1, 3, and 7 after intervention with a STING agonist (ADU-S100) or inhibitor (C-176) are shown in Fig. 5 H. N=3; Error bars, mean ± SD, * P<0.05, * * P<0.01.

**Appendix Table**

**Table S1 Antibodies of proteins used in western blot and immunofluorescence analyses**

| **REAGENT or RESOURCE** | **SOURCE** | **IDENTIFIER** |
| --- | --- | --- |
| **Antibodies** | | |
| Rabbit monoclonal anti – Piezo1 | Thermo Fisher Scientific | Cat# PA5-106296 |
| Rabbit monoclonal anti - alpha Tubulin | Abcam | Cat# ab176560 |
| Rabbit monoclonal anti - CDKN2A/p16^INK4a^ | Abcam | Cat# ab270058 |
| Mouse monoclonal anti - IP3R-I/II/III | Santa Cruz | Cat# sc-377518 |
| Rabbit monoclonal anti - VDAC1 | Affinity | Cat# AF4012 |
| Mouse monoclonal anti - CBARA1/MICU1 | Santa Cruz | Cat# sc-518183 |
| Rabbit monoclonal anti - Hexokinase II | Abcam | Cat# ab209847 |
| Rabbit monoclonal anti - BAX | Abcam | Cat# ab32503 |
| Mouse monoclonal anti - dsDNA | Progen | Cat# 61014 |
| Rabbit monoclonal anti - TOMM20 | Abcam | Cat# ab186735 |
| Rabbit monoclonal anti - cGAS | Abcam | Cat# ab302617 |
| Rabbit monoclonal anti - STING | Abcam | Cat# ab288157 |
| Rabbit monoclonal anti - TBK1 | Abcam | Cat# ab40676 |
| Rabbit monoclonal anti - p-TBK1 | Abcam | Cat# ab109272 |
| Rabbit monoclonal anti -IRF3 | Abcam | Cat# ab68481 |
| Rabbit monoclonal anti - p-IRF3 | Abcam | Cat# ab76493 |
| Rabbit monoclonal anti - NF-κB | Abcam | Cat# ab16502 |
| Rabbit monoclonal anti - p-NF-κB | Abcam | Cat# ab109458 |
| Rabbit monoclonal anti - IL-1β | Abcam | Cat# ab315084 |
| Rabbit monoclonal anti - IL-6 | Abcam | Cat# ab9324 |

**Table S2. List of siRNA used in the study.**

| **Primer** | **Sequence (5'to3 ')** |
| --- | --- |
| ITPR3(human)siRNA-2836 | CCAACGAGGAGAAGAACAATT |
|  | UUGUUCUUCUCCUCGUUGGTT |
| ITPR3(human)siRNA-3709 | CGCCAAGGACAAGAAAGATT |
|  | UCUUUCUUGUCCUUGGCGGTT |
| ITPR3(human)siRNA-3583 | CGCAGGACGUGGAGAACUATT |
|  | UAGUUCUCCACGUCCUGCGTT |
| Negative control | UUCUCCGAACGUGUCACGUTT |
|  | ACGUGACACGUUCGGAGAATT |
| FAM negative control | UUCUCCGAACGUGUCACGUTT |
|  | ACGUGACACGUUCGGAGAATT |
| Positive control (human GAPDH) | GUAUGACAACAGCCUCAAGTT |
|  | CUUGAGGCUGUUGUCAUACTT |

**Table S3.** **List of primer used in the study**

| **Primer** | **Sequence (5'to3 ')** |
| --- | --- |
| Piezo1 | TGTTCCAGTTTGGGTTCTTCC |
|  | ACAAGGTCAAACCCAAGAAGG |
| IL-1β | GAAATGATGGCTTATTACAGTGGC |
|  | TTGCTGTAGTGGTGGTCGGAG |
| IL-6 | GAGTAGTGAGGAACAAGCCAGAG |
|  | GGTCAGGGGTGGTTATTGC |
| MMP3 | CAATCCTACTGTTGCTGTGCG |
|  | CAAGGTTCATGCTGGTGTCC |
| MMP14 | GCCTGCGTCCATCAACACT |
|  | GCAGCATCAATCTTGTCGGT |
| CXCL3 | CCAAACCGAAGTCATAGCCAC |
|  | GGTGCTCCCCTTGTTCAGTATC |
| CXCL10 | TGACTCTAAGTGGCATTCAAGGA |
|  | TGGATTCAGACATCTCTTCTCACC |
| AREG | AGTGCTGATGGATTTGAGGTTAC |
|  | TAGCCAGGTATTTGTGGTTCG |
| EREG | GCCTGGGTTTCCATCTTCTAC |
|  | CACTTTGTTATTGACACTTGAGCC |
| ITPR3 | GACCTCCTGTTCTTCTTCATCG |
|  | TGTCTTGTTATCAAACTTGTCCCTC |
| VDAC1 | TGACGGGCAGTCTGGAAAC |
|  | ATCTCGGTGCCTAGTGTATTGTC |
| MICU1 | TGGCGAACTGAGCAATAAGG |
|  | GCATGAGGCGAGTGAAACC |
| BAX | CCTCCTCTCCTACTTTGGGAC |
|  | GAAAAACACAGTCCAAGGCAG |
| ACTB | AGAAAATCTGGCACCACACCT |
|  | GATAGCACAGCCTGGATAGCA |
| 18sRNA | GGACACGGACAGGATTGACAG |
|  | CCAGAGTCTCGTTCGTTATCGG |
| MT-COL1 | ACTAACAGACCGCAACCTCAAC |
|  | TCCGAAGCCTGGTAGGATAAG |
| MT-ND1 | CACCCTCACCACTACAATCTTCC |
|  | GGTGTATGAGTTGGTCGTAGCG |
| MT-ND2 | AACCCGTCATCTACTCTACCATC |
|  | GTTGCTTGCGTGAGGAAATAC |
| cGAS | CGTATGTACCCAGAACCCTCA |
|  | TGTCACGCAGTTATCAAAGCAG |
| STING | TGATAACCTGAGTATGGCTGACC |
|  | TGATAACCTGAGTATGGCTGACC |
| TBK1 | TCTCAGGGAACAATAGAAACCAG |
|  | GGATGAGTGCCTTCTTGATGTG |
| IRF3 | GGCTCGTGATGGTCAAGGT |
|  | CTTGTACTGGTCGGAGGTGAG |
| NFKB-RELA | TGGGGACTACGACCTGAATG |
|  | AAGATGGGATGAGAAAGGACAG |
